# Desymmetrization Approach to the Synthesis of Optically Active P-Stereogenic Phosphin-2-en-4-ones

**DOI:** 10.1021/acs.joc.0c03055

**Published:** 2021-04-26

**Authors:** Elżbieta Łastawiecka, Sławomir Frynas, K. Michał Pietrusiewicz

**Affiliations:** Department of Organic Chemistry, Institute of Chemical Sciences, Faculty of Chemistry, Marie Curie-Sklodowska University, Gliniana 33 St., 20-614 Lublin, Poland

## Abstract

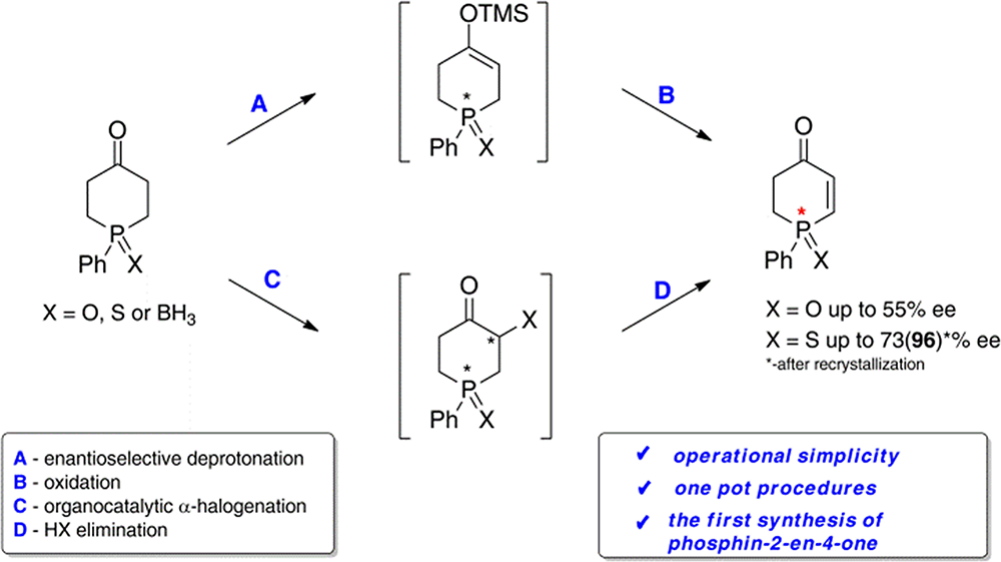

Two synthetic protocols for the conversion of 1-phenylphosphinan-4-ones to novel P-stereogenic 1-phenylphosphin-2-en-4-ones by enantioselective deprotonation followed by oxidation and by asymmetric organocatalytic halogenation accompanied by elimination have been developed. These two-step one-pot transformations provide convenient access to optically active 1-phenylphosphin-2-en-4-one 1-sulfide and 1-phenylphosphin-2-en-4-one 1-oxide of 96 and 55% enantiomeric purities, respectively.

## Introduction

**Figure 1:**
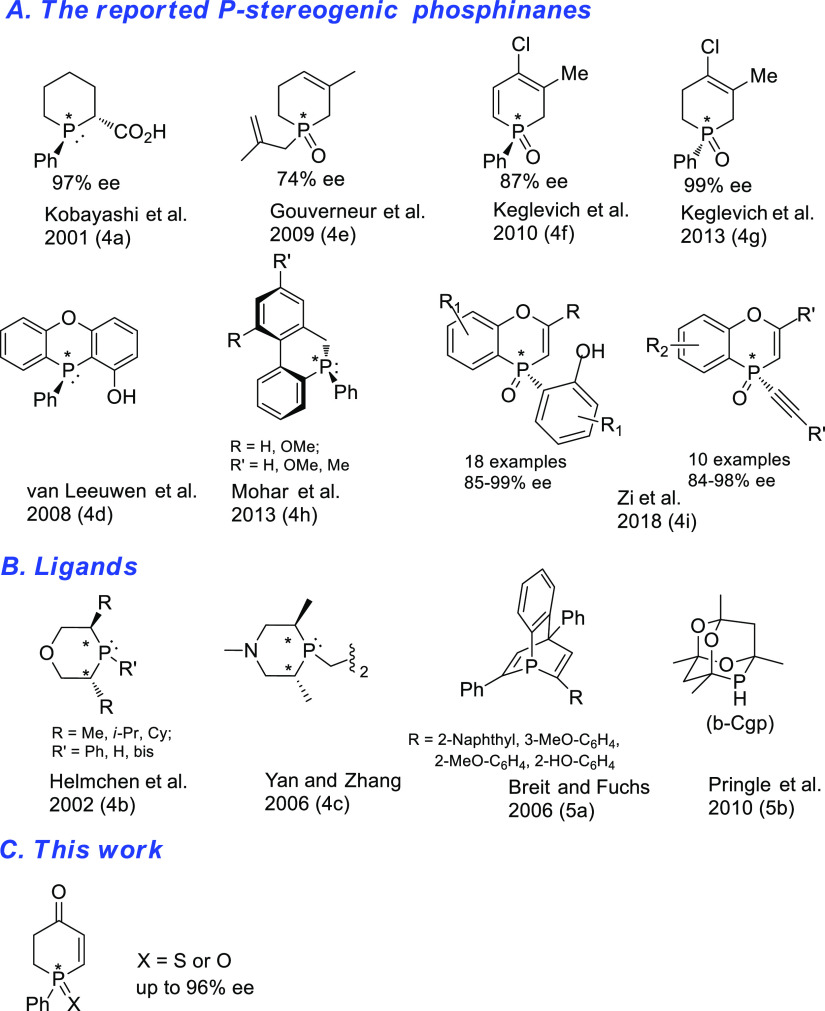
Reported optically active phosphinanes.

Cyclic nonracemic phosphines constitute an important group of organophosphorus compounds that are sought for their advantageous performance as organocatalysts and as ligands in various asymmetric processes.^1^ Numerous chiral five-membered (phospholane)^2^ and four-membered (phosphetane) ligands^3^ have been developed to meet the demand. In contrast, the corresponding chiral six-membered carbon-phosphorus heterocycles (phosphinanes) have received relatively little attention^4,5^ due, most probably, to scarcity of convenient methods enabling their synthesis in suitably functionalized and nonracemic forms.^6^ For illustration, all the optically active phosphines and phosphine oxides containing phosphorus embedded in the six-membered ring, which have been synthesized to date, are collected in Figure 1A,B.

There has recently been considerable interest in preparation of P-stereogenic phosphorus compounds by desymmetrization reactions starting from P-prochiral precursors.^7^ Synthesis of cyclic phosphine derivatives by this route can start either from an acyclic,^4e^^4i^ or from a cyclic^8,9^ precursor. In the latter case, the reported precedents included phosphol-3-ene oxide^8^ and its epoxide,^9^ phosphetane sulfide,^3d^ and phospholane sulfide^2f^ as well as phospholane borane and phosphinane boranes.^4a^ In this paper, we wish to report our results on evaluation of enantioselective desymmetrization of 1-phenylphosphinan-4-one (**1**) by employing its carbonyl function in two independent two-step processes designed to lead to the formation of optically active 1-phenylphosphin-2-en-4-one derivatives **4** (Figure 1C). The target phosphin-2-en-4-one, equipped with a versatile enone functionality, represents a novel phosphinane scaffold potentially amenable to rich chemistry further downstream.

## Results and Discussion

**Scheme 1:**
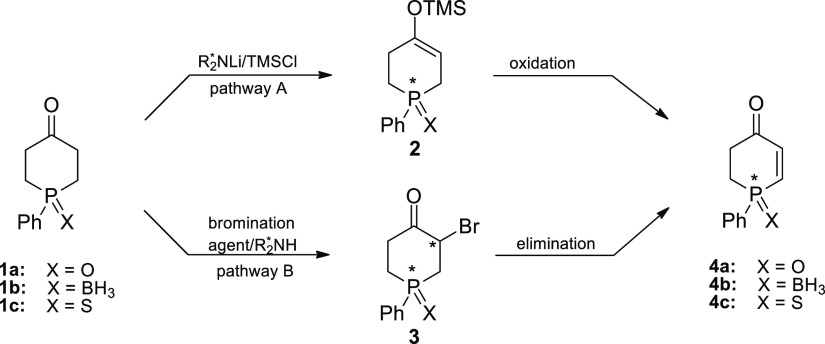
Designed Desymmetrization Routes to 1-Phenylphosphin-2-en-4-ones **4**

Of the known synthetic methods used frequently for desymmetrization of prochiral ketones,^10^ enantioselective deprotonation,^10a^^10b^ and enantioselective α-halogenation,^10f^^10g^ seemed to be most suitable for accomplishing our goal. Accordingly, the two alternative paths that we have designed to lead to optically active **4** are based on these two desymmetrization processes (Scheme 1).

The desymmetrization by path A involves asymmetric deprotonation of 1-phenylphosphinan-4-one (**1**) by a chiral base and conversion of the resulting lithium enolate to the silyl enol ether **2** by quenching with TMSCl.^10a^^10b^ The desymmetrization by path B entails transformation of phosphinanone **1** into a chiral α-halogenated derivative **3**, which could be achieved by organocatalytic asymmetric α-halogenation.^10f^^10g^ Both synthetic procedures make use of the ketone functionality of phosphinanone **1**, and both result in the overall asymmetric transformation of the remote prochiral phosphorus center in ketone **1** into a P*-*stereogenic one in enone **4** via intermediate **2** or **3**.

Since the time the enantioselective deprotonation of cyclic ketones by a chiral lithium amide was first demonstrated in 1986,^10a^^10b^ the method has been widely utilized in asymmetric synthesis for generating chirality centers in cyclic ketones by desymmetrization.^11^ Although efficient desymmetrizations of a number of oxa-, aza-, and thia-heterocyclic ketones by chiral lithium amides have been already demonstrated,^10f^^10^^12^ the corresponding P-heterocyclic analogs have not been investigated before. Thus, we started with checking the viability of enantioselective deprotonations of 1-phenylphosphinan-4-one 1-oxide (**1a**), 1-borane (**1b**), and 1-sulfide (**1c**) using lithium amide derived from amine (*S*,*S*)-**5** as the model base premixed with an excess of TMSCl before addition of a ketone (ISQ - in situ quench)^13^ (Table 1).

**Table 1:**
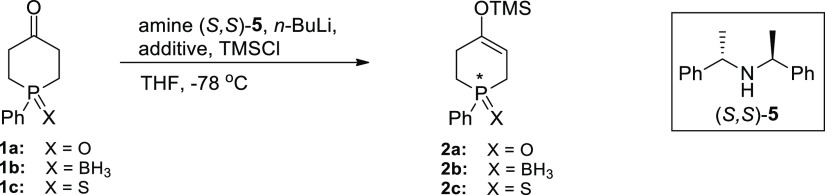
Enantioselective Deprotonation of **1** Using Lithium Amide Derived from Amine **5**^a^

no.	X	procedure	additive (equiv.)	yield [%]^b^	ee [%]^c^
1	O	ISQ		traces	n.d.
2	BH_3_	ISQ		17	7
3	S	ISQ		18	8
4	S	ISQ	HMPA (2)	19	4
5	S	ISQ	LiCl (0.5)	71	53
6	S	EQ	LiCl (0.5)	76	54
7	S	EQ	LiCl (1)	81	52

a Standard reaction conditions: **1** (0.1 mmol), (*S*,*S*)-**5** (0.3 mmol, 3 equiv.), *n*-BuLi (1.6 M solution, 3 equiv.), TMSCl (0.5 mmol, 5 equiv.), in THF (2 mL; *c* = 0.05 mol/L), at −78 °C for 1 h.

b Determined by ^31^P NMR.

c Determined for the crude reaction mixture by CSP-HPLC.

As shown in Table 1 (entries 1–3), phosphinanone oxide **1a** failed to provide silyl enol ether **2a**, whereas borane **1b** and sulfide **1c** gave the expected enol ethers **2b** and **2c**, respectively, albeit in low yields and with very low ee. Subsequent testing of phosphinanone sulfide **1c** revealed that addition of 0.5 equiv. of LiCl allowed increasing the yield and ee of silyl enol ether **2c** to more acceptable levels (entry 5) and that allowing lithium amide to react with phosphinanone sulfide **1c** before TMSCl was introduced (EQ - external quench)^14^ gave slightly better results than the ISQ alternative (entries 5 and 6). As checked under these conditions again, the amount of 0.5 equiv. of LiCl was sufficient; increasing its loading to 1 equiv. did not bring about improvement of ee.

The details of further optimization of these reaction conditions, which included variations of molarity, stoichiometry, and temperature, are presented in Table 2.

**Table 2:**
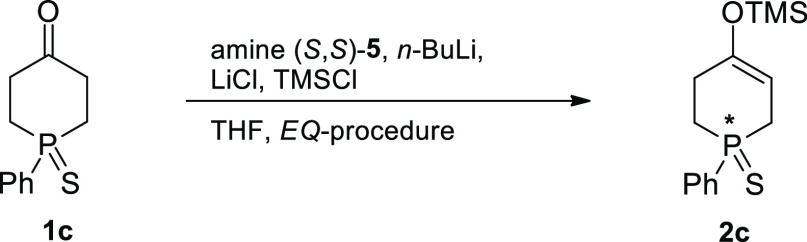
Optimization of Conditions of Enantioselective Deprotonation of Phosphinanone Sulfide **1c**^a^

no.	amine (equiv.)	*n*-BuLi (equiv.)	*C*_m_ [mol/dm^3^]	temp. [^o^C]	yield [%]^b^	ee [%]^c^
1	1.5	1.5	**0.05**	–78	95	12
2	1.5	1.5	**0.025**	–78	63	54
3	1.5	1.5	**0.016**	–78	18	61
4	**2**	1.5	0.025	–78	98	67
5	**3**	1.5	0.025	–78	85	74
6	3	3	0.025	**–78**	75	59
7	3	3	0.025	**–20**	40	0
8	3	3	0.025	**–90**	77	83
9^d^	3	3	0.025	**–90**	87	86
10	3	1.5	0.025	**–90**	81	**87**
11^d^	3	1.5	0.025	**–90**	85	86
12	3	1.5	0.016	**–90**	51	87

a Standard reaction conditions: **1** (0.1 mmol), (*S*,*S*)-**5**, *n*-BuLi (1.6 M solution), LiCl (0.05 mmol), TMSCl (0.5 mmol, 5 equiv.), in THF for 1 h.

b Determined by ^31^P NMR.

c Determined for the crude reaction mixture by CSP-HPLC.

d Reaction run with 1 equiv. of LiCl.

As shown in Table 2, lowering of concentration led to improvement of enantioselectivity, but unfortunately, it led to a substantial decrease in yield (entries 1–3). The concentration of 0.025 M was deemed a practical compromise and was then used in subsequent trials. A substantial increase of enantioselectivity to 74% ee at 85% conversion was observed when 3 equiv. of amine **5** was used instead of 1.5 equiv. (entries 4 and 5). In addition, lowering of the reaction temperature to −90 °C resulted in further enhancement of enantioselectivity up to 87% ee at 81% conversion (entry 10). Finally, checking the concentration factor once again confirmed that its lowering resulted in a substantial decrease in yield, but this time, it was not even accompanied by an increase of enantioselectivity observed before (cf. entries 10 and 12).

Once the optimization of the reaction conditions was completed, also other amine catalysts were tested for their efficiency in desymmetrization of 1-phenylphosphinan-4-ones **1c** and **1b**. The results obtained with chiral monoamines **5**–**13**, **15**, and **16** and diamines **14** and **17–19** are displayed in Table 3.

**Table 3:**
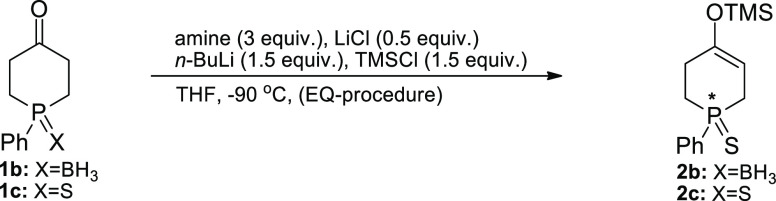

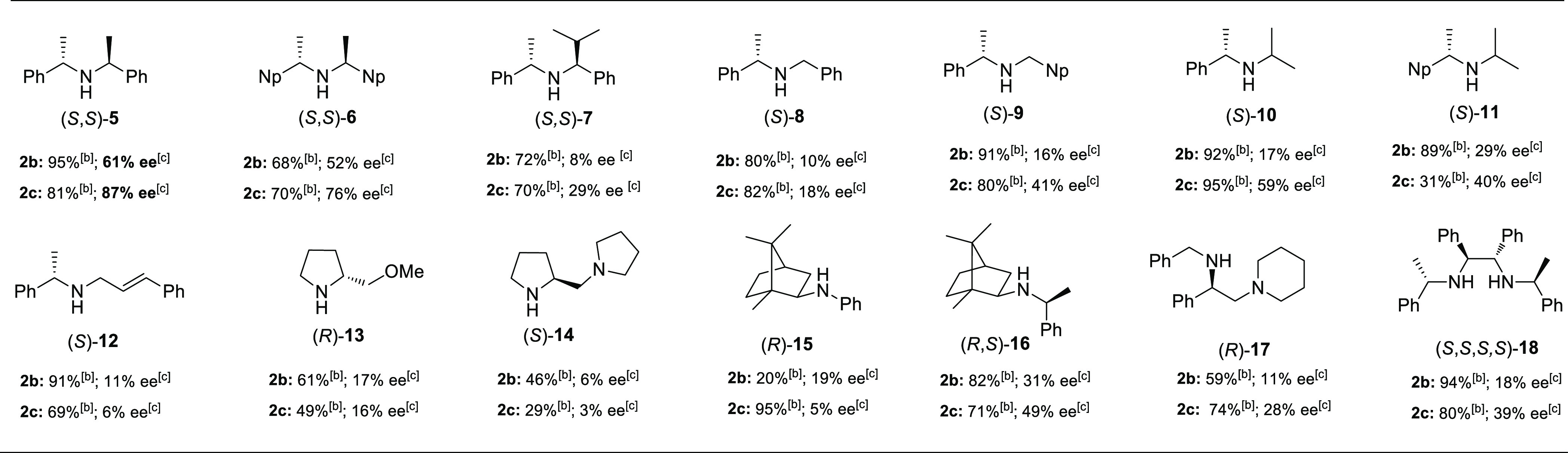
Screening of Chiral Amines **5**–**19** in Desymmetrization of Phosphinanones **1b**,**c** by Enantioselective Deprotonation under Optimized Conditions^a^

a Standard reaction conditions: **1** (0.1 mmol), chiral amine **5–18** (0.3 mmol, 3 equiv.), *n*-BuLi (1.6 M solution, 1.5 equiv.), THF (4 mL; *c* = 0.025 mol/L), at −78 °C for 1 h, TMSCl (0.5 mmol, 5 equiv.).

b Determined by ^31^P NMR.

c Determined for the crude reaction mixture by CSP-HPLC.

Inspection of the results collected in Table 3 reveals that the best enantioselectivities in desymmetrization of phosphinanone sulfide **1c** were achieved with *C*_2_-symmetric lithium bis(α-arylethyl)amides derived from amines (*S*,*S*)-**5** and (*S*,*S*)-**6**, i.e., 87 and 76% ee, respectively. The *C*_1_-symmetric α-phenylethylamine derived bases **7**–**12** and **16** were also effective in desymmetrizing phosphinanone sulfide **1c** and gave silyl enol ether **2c** in good yield and with enantioselectivity reaching 59% ee. Diamines **14** and **17**–**19** gave slightly lower enantioselectivities than the monoamines. In turn, desymmetrization of phosphinanone borane **1b** carried out with lithiated **5**–**19** under the same conditions gave silyl enol ether **2b** in generally better yields but with much lower enantioselectivities than sulfide **2c**. For borane **2b**, the best ee’s were again achieved with lithium amides derived from (*S*,*S*)-**5** and (*S*,*S*)-**6**, i.e., 61% ee at 95% conversion and 52% ee at 68% conversion, respectively.

Next, we turned our attention to the oxidation of silyl enol ethers **2b**,**c** required for their conversion into phosphinenones **4b**,**c**. Our initial attempts involved use of the well-known procedures utilizing Pd(OAc)_2_ in acetonitrile,^15^ DDQ in benzene, and trityl tetrafluoroborate in dichlorometane^16^ as the oxidizing agents, but with these reagents, phosphinenones **4** were produced in very low yields (Table 4, entries 1–3).

**Table 4:**
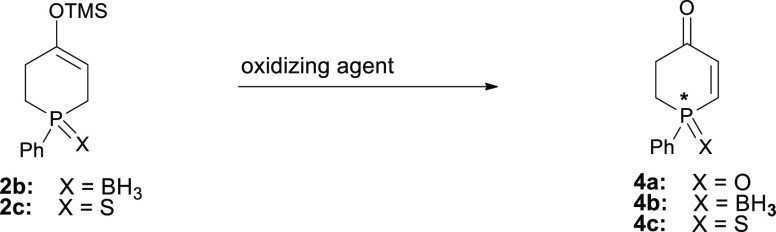
Oxidation of Silyl Enol Ethers **2b**,**c**

no.	X	oxidizing agent (equiv.)	solvent	conditions	ratio^a^ (enone **4**/ketone **1**)
1	S	Pd(OAc)_2_ (1)	CH_3_CN	rt,^d^ 10 h	7/93
2	S	DDQ (1.5)	C_6_H_6_	rt, 1 h	1/99
3	S	PhC^+^BF_4_^–^ (2)	DCM	rt, 12 h	5/95
4	S	CAN (2.5)	DMF	0 °C → rt, 2.5 h	69/31
5	BH_3_	CAN (2.5)	DMF	0 °C → rt, 2.5 h	74/26^b^
6	S	IBX (3)	DMSO	40 °C, 12 h	21/79
7	BH_3_	IBX (3)	DMSO	40 °C, 12 h	19/81^b^
8	S	IBX·MPO (4)	DMSO	rt, 2 h	**80/20**(67)^c^
9	BH_3_	IBX·MPO (4)	DMSO	rt, 2 h	**73/27**^b^(58)^c^

a Determined for the crude reaction mixture by GC–MS and ^31^P NMR analysis.

b Identified as oxides **4a** and **1a** due to P-oxidation occurring under the reaction conditions.

c Isolated yield of enone **4**.

d rt = 18–22 °C.

Subsequent treatment of silyl enol ethers **2c** and **2b** with ceric ammonium nitrate (CAN) in DMF^17^ led to the formation of phosphinenones **4c** and **4a** in 69 and 74% yields, respectively (entries 4 and 5). It should be noted, however, that under these conditions, phosphinenone borane **4b** could not be obtained due to concurrent oxidation of the P center during the reaction course. Finally, using Nicolaou et al.’s procedure for the oxidation of silyl enol ethers to α,β-unsaturated carbonyl compounds utilizing the IBX·MPO complex as the oxidant,^18^ phosphinenones **4c** and **4a** (from **2b**) were obtained in high yields, 80 and 73%, respectively (entries 8 and 9).

**Scheme 2:**
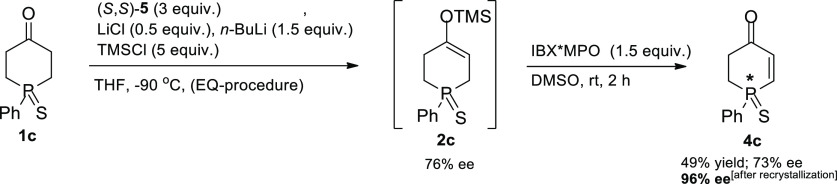
One-Pot Synthesis of Phosphinenone **4c** on a Preparative 1.1 g (5 mmol) Scale

Encouraged by the latter’s promising results and taking into account the fact that silyl enol ethers **2c** and **2b** proved to be highly susceptible to hydrolysis during chromatographic purification, we decided to combine the best desymmetrization and oxidation protocols found for phosphinanone **1c** in a one-pot process to avoid substantial loss of the intermediate silyl enol ether during isolation (Scheme 2).

As shown in Scheme 2, the two steps carried out in one flask without isolation of the intermediate **2c** furnished phosphinenone **4c** in overall 49% isolated yield. The determination of enantiomeric excesses of intermediate silyl enol ether **2c** and of the obtained phosphinenone **4c** (CSP-HPLC) revealed that a slight loss of enantiomeric purity might have taken place during the oxidation step. Importantly, however, recrystallization of the isolated sulfide **4c** of 73% ee from hexane/*i*-PrOH allowed its enantiomeric purity to increase to 96% ee.

In the second part of our study, we turned our attention to another organocatalytic strategy expected to be suitable to achieve our goal. In 2005, Jørgensen et al.^10g^ described the first enantioselective α-bromination of ketones utilizing *N*-bromosuccinimide (NBS) and 4,4-dibromo-2,6-di-*tert*-butyl-cyclohexa-2,5-dienone (**20**) as the brominating agents and (*S*)-proline and a *C*_2_-symmetric imidazolidine as the chiral catalysts. These reagents enabled the formation of stereogenic C–Br centers with up to 94% ee in high yields.^10g^ We decided then to check the viability of this protocol in the asymmetric α-bromination of phosphinanones **1a**,**c**, which, when followed by elimination of HBr, could lead to the target optically active phosphinenones **4a**,**c**.

We started our investigations with a brief screening of solvents and additives in α-bromination of oxide **1a** and sulfide **1c**, using NBS (or **20**) as the brominating agent and (*S*)-proline as the model chiral catalyst. At the outset, we were pleased to find that elimination of HBr started to occur already under the bromination conditions and that practically quantitative elimination of HBr could be achieved by simply raising the temperature at the end of the reaction to 60 °C for half an hour. We included this maneuver to the screening conditions to make the planned synthesis of phosphinenones **4a**,**c** a one-pot process (Table 5).

**Table 5:**
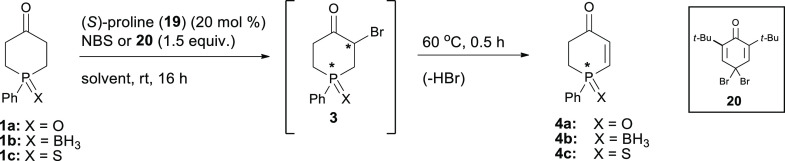
Preliminary Screening of Reaction Conditions for Conversion of Phosphinanones **1a**–**c** to Phosphinenones **4a**–**c** via Catalytic α-Bromination^a^

no.	X	solvent	additive (20 mol %)	Br-source	yield **4a**,**c** [%]^b^	ee [%]^c^
1	O	DCM		NBS	45	10
2	O	THF		NBS	74	1
3	O	DMF		NBS	61	8
4	O	CH_3_CN		NBS	41	6
5	O	DCM	AcOH	NBS	42	32
6	O	DCM	PhCOOH	NBS	47	34
7	O	DMF	PhCOOH	NBS	21	6
8	O	DCM		NBS	76(64)^d^	
9	O	DCM	PhCOOH	20	47	11
10	BH_3_	DCM	PhCOOH	NBS		
11	BH_3_	DCM	PhCOOH	20	traces	
12	S	DCM	PhCOOH	NBS		
13	S	DCM	PhCOOH	20	57	24

a Standard reaction conditions: NBS (0.15 mmol) was added to a mixture of **1** (0.1 mmol), an additive (20 mol %), and amine catalyst (20 mol %) in the indicated solvent (2 mL) and stirred at room temperature for 16 h and at 60 °C for 0.5 h.

b Determined by GC–MS and ^31^P NMR analysis.

c Determined for the crude reaction mixture by CSP- HPLC.

d Yield of the isolated product in parentheses.

As can be seen from the collected data, a change of solvent as well as an added acid^19^ can strongly influence the outcome of the reaction (Table 5, entries 7–13). With added benzoic acid, the enantiomerically enriched **4a** was obtained with 34% ee and in 47% yield, what constituted a significant improvement over the reaction run without this additive in the same solvent (DCM) (cf. entries 7 and 12). In turn, changing the solvent to THF or DMF resulted in a marked increase of the conversion, but the observed enantioselectivity was significantly lowered. Thus, the conditions utilizing DCM and added benzoic acid (entry 12), which best compromised the conversion and induction levels, were selected for screening of a number of other chiral amine catalysts in the next optimization step. The results of this screening are summarized in Table 6. The reactions of all tested amines were performed with and without benzoic acid, but only the better result of these two runs has been listed for clarity.

**Table 6:**
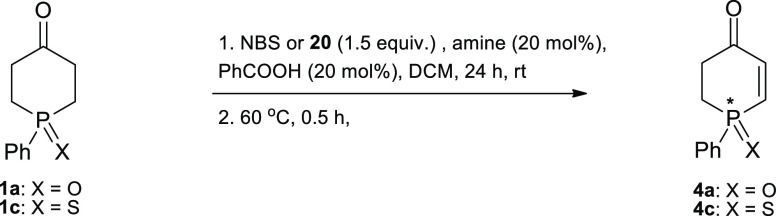

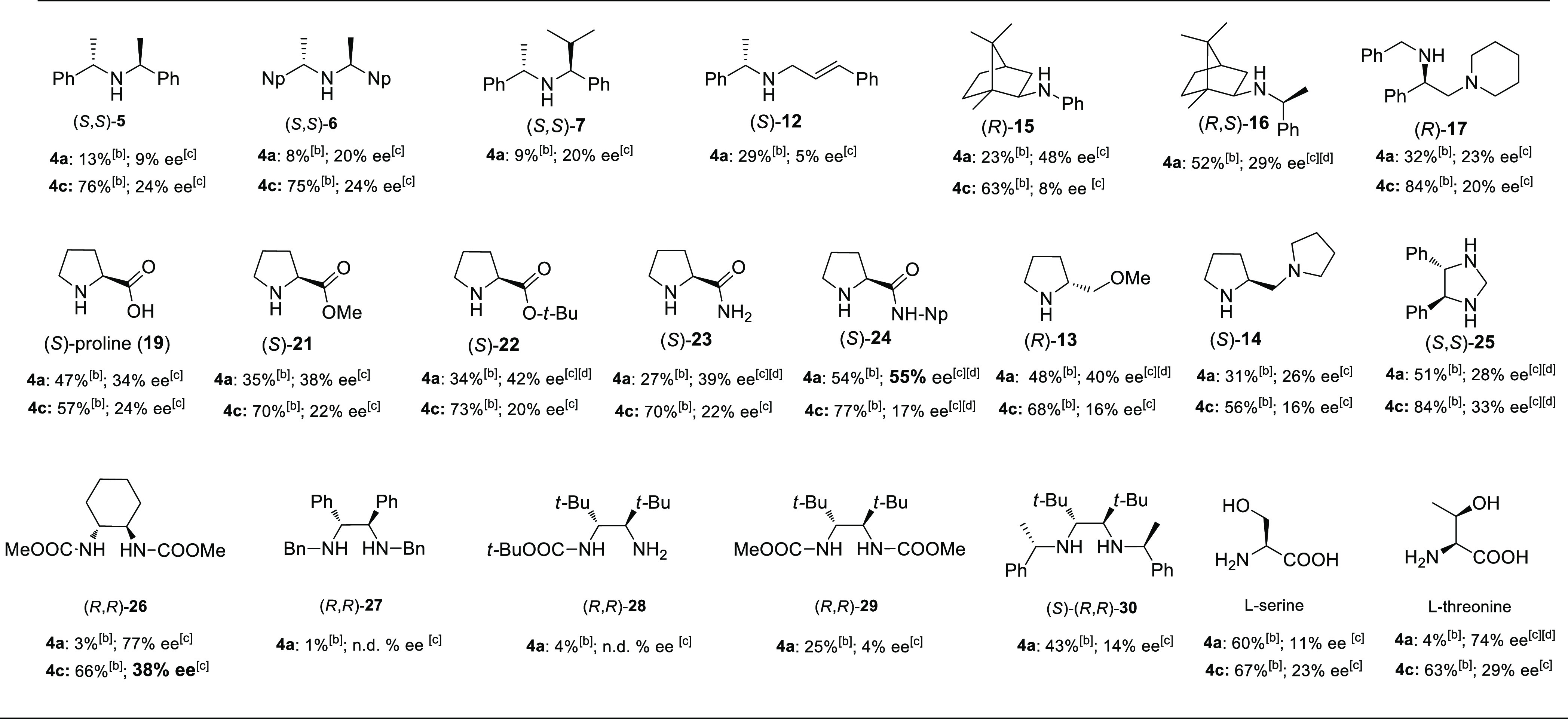
Evaluation of Amine Catalysts in Conversion of Phosphinanones **1a**,**c** to Phosphinenones **4a**,**c** via Enantioselective α-Bromination under Optimized Conditions*^a^*

a Procedure: To a mixture of **1a** or **1c** (0.1 mmol), PhCOOH (0.02 mmol), and a catalyst (0.02 mmol) in DCM (2 mL), NBS or **20** (0.15 mmol) was added and the reaction mixture was stirred at room temperature for 16 h and then at 60 °C for 0.5 h.

b Yields of **4a** and **4c** determined by GC–MS analysis.

c Enantiomeric excess determined for the crude reaction mixture by CSP-HPLC.

d Reaction run without PhCOOH.

As can be seen in Table 6, screening of amines **13**–**17** and **21**–**24** as the organocatalysts allowed the enantioselectivity of bromination of phosphinanone **1a** to increase only up to 55% ee when (*S*)-proline naphthylamide **24** was used as the catalyst. Interestingly, *C*_2_-symmetric 4,5-diphenyl-imidazolidine (**25**), the reported most efficient catalyst for enantioselective α-bromination of cyclic ketones,^10g^ afforded enone **4a** of only 28% ee. Apparently, pyrrolidine based amines performed somewhat better than other amines tested in the studied α-bromination of phosphinanone **1a**. Surprisingly inefficient were *C*_2_-symmetric diamines even though DACH-derived (*R*,*R*)-**26** afforded enone **4a** of 77% ee but, unfortunately, at nearly negligible 3% conversion.

Also listed in Table 6 are the results of desymmetrization of phosphinanone sulfide **1c** carried out with compound **20** as the brominating agent under otherwise the same conditions. These reactions proceeded relatively well and afforded enone **4c** in good yields (56–84%) but with only moderate enantiomeric enrichment (8–38% ee). Possibly the best match of yield and enantiomeric purity of **4c** was achieved with DACH derivative (*R*,*R*)-**26** (66% and 38% ee, respectively) and with imidazolidine (*S*,*S*)-**25** (84% and 33% ee, respectively).

Looking for further improvement, we also decided to briefly check the efficiency of analogous enantioselective α-chlorinations, which have been recently demonstrated to be highly efficient in the case of six-membered-ring ketones.^10f^ The results of screening experiments involving chlorination of phosphinanones **1a**,**c** by NCS and PhICl_2_ in the presence of (*S*)-proline and other amine catalysts, followed by DBU-assisted elimination of HCl from intermediate α-chloro ketone **3-Cl** to give enone **4a**,**c**, are collected in Table 7.

**Table 7:**
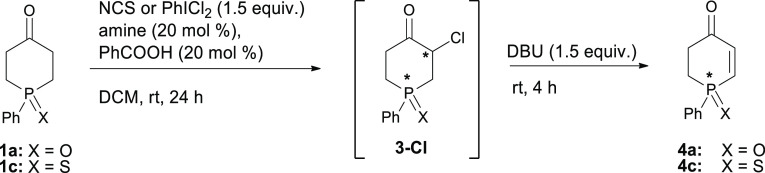
Synthesis of Phosphinenones **4a**,**c** via Organocatalytic Enantioselective α-Chlorination of **1a**,**c**^a^

no.	X	catalyst	Cl-source	yield **4a**,**c** [%]^b^	ee [%]^c^
1^c^	O	(*S*)*-***19**	NCS	24	*rac*
2	O	(*S*)-**19**	NCS	3	*rac*
3^c^	O	(*S*)-**19**	PhICl_2_	26	*rac*
4	O	(*S*)-**19**	PhICl_2_	56	*rac*
5	O	(*S,S*)-**25**	NCS	34	30
6	O	(*S,S*)-**25**	PhICl_2_	74	21
7	S	(*S,S*)-**25**	PhICl_2_	0	
8	O	(*R*)-**17**	PhICl_2_	91	*rac*
9	O	(*S*)-**21**	PhICl_2_	87	*rac*
10	O	(*S*)-**24**	PhICl_2_	88	*rac*

a Standard reaction conditions: NCS or PhICl_2_ (0.15 mmol) was added to a mixture of of **1** (0.1 mmol), PhCOOH (20 mol %), and amine catalyst (20 mol %) in DCM (2 mL) and stirred at rt for 1 day. DBU (1.5 equiv.) was then added, and the reaction mixture was stirred at rt for an additional 4 h to effect elimination of HCl.

b Determined by ^31^P NMR spectroscopy.

c Enantiomeric excess determined for the crude reaction mixture by CSP-HPLC.

The collected data reveal that PhICl_2_ as the chlorine source gave better conversions than NCS and that addition of benzoic acid had a beneficial effect on the overall yield of phosphinenone **4a**, especially in combination with PhICl_2_. Under these conditions, (*S*)-proline catalyzed the formation of α-chlorophosphinanone intermediate **3-Cl** in moderate yields but, unfortunately, the resulting enone **4a** was formed as a racemate (entries 1–4). Similarly, chlorinations of phosphinanone **1a** with amines **17**, **21**, and **24** as the catalysts also led to the formation of racemic enone **4a**, although in these cases with remarkably high conversions of 91, 87, and 88%, respectively (entries 8–10). In turn, imidazolidine (*S*,*S*)-**25**, the reported excellent catalyst for the asymmetric α-chlorination of six-membered-ring ketones,^10f^ afforded enantioenriched enone **4a** of only 30 (with PhICl_2_) or 21% ee (with NCS) in moderate 34% and good 74% yields, respectively (entries 5 and 6). It is important to note, however, that in these two cases, as determined by comparison of the pertinent CSP-HPLC chromatograms, the use of (*S*,*S*)-**25** as the catalyst led to the formation of enone **4a** enriched in the enantiomer opposite to that found in predominance in **4a** obtained by the α-bromination procedure utilizing the same (*S*,*S*)-**25** as the catalyst. Interestingly, an attempted reaction of sulfide **1c** under exactly the same conditions failed completely (entry 7). At this point, considering that the prospect of getting high enantioselectivity in desymmetrizations of phosphinanones **1a**,**c** by α-chlorination did not look promising, further optimization of this process was discontinued. Nonetheless, despite the fact that the chlorination procedure did not provide the expected improvement of enantioselectivity in the studied syntheses of optically active phosphinenones **4**, the developed one-pot chlorination-elimination procedure is likely to find use as an effective method for synthesis of racemic phosphinenone oxide **4a** (cf. entries 8–10).

**Scheme 3:**
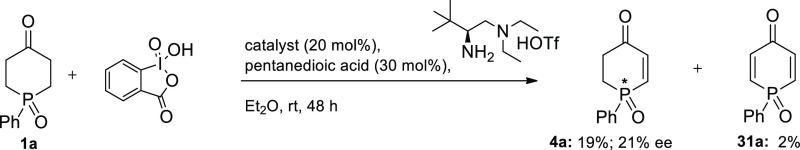
Attempted Enantioselective Desymmetrization of Phosphinanone **1a** through Enamine Oxidation^20^

All in all, it is tempting to conclude that enantioselective α-halogenation of phosphinanone **1**, a six-membered-ring ketone possessing a phosphorus function in the γ position, is considerably more challenging than the parent cyclohexanone and related six-membered-ring ketones.,^10f^^10g^ Moreover, a poor result of our attempted organocatalytic desymmetrization of phosphinanone oxide **1a** via enamine oxidation under recently reported optimized conditions^20^ shown to be effective in converting a whole variety of mono and doubly 4-substituted cyclohexanones to the corresponding cyclohexenones of very high enantiomeric purity corroborates this notion further (Scheme 3).

## Conclusions

Even though the asymmetric deprotonation and asymmetric halogenation of phosphinanone **4** have turned out to be less efficient than those of carbocyclic ketones, the developed one-pot enolization-oxidation and halogenation-elimination procedures have for the first time provided access to the new P-stereogenic phosphin-2-en-4-one derivatives in nonracemic forms. A good level of asymmetric induction (87% ee at 81% conversion) can be achieved by enantioselective deprotonation of phosphinanone sulfide **1c** at −90 °C using 3 equiv. of lithium amide derived from commercially available amine *S*,*S*-**5**. Subsequent in situ oxidation of the formed enantiomerically enriched silyl enol ether **2c** by IBX·MPO converts it to optically active phosphinenone **4c**, the enantiopurity of which can be upgraded to 96% ee by recrystallization. Desymmetrization of phosphinanone oxide **1a** can be best achieved by asymmetric α-bromination using (*S*)-proline amide **24** as the catalyst to provide enriched 3-bromophosphinanone **3**, which, in turn, undergoes in situ elimination of HBr to afford phosphinenone **4a** of 55% ee in 54% yield. The analogous asymmetric α-chlorination-elimination procedure offers very low or even no enantioselectivity in desymmetrization of phosphinanone **1a**. Nevertheless, it allows obtaining phosphinenone oxide **4a** in very high yields (cf. Table 7, entries 8–10) and may thus constitute a useful route to *rac*-**4a**.

## Experimental Section

### General Information

All reactions were performed under an argon atmosphere using Schlenk techniques or in a 10 mL glass reaction tubes with a screw cap. Only dry solvents were used, and the glassware was heated under vacuum prior to use. THF was dried over sodium/benzophenone ketyl. LiCl was dried in a Schlenk tube under vacuum at 150 °C for 5 h. TMSCl, NBS, MPO, DMSO, chiral amines **5**, **6**, **13**, **14**, **21**, and (*S*)-proline (**19**) were purchased from commercial sources and used without further purification. Solvents for chromatography and extraction were commercially available and used as received without further purification. Solvents for crystallization and Et_3_N were distilled once before use. Room temperature (rt) means a range of temperatures from 18 to 22 °C.

The NMR spectra were recorded with a Bruker Ascend (500 MHz) spectrometer in CDCl_3_ as a solvent at room temperature unless otherwise noted. Chemical shifts (δ) are given in ppm relative to tetramethylsilane (^1^H), residual CHCl_3_ (^13^C), or external 85% H_3_PO_4_ (^31^P) as a reference. The following abbreviations are used in reporting NMR data: s (singlet), d (doublet), t (triplet), q (quartet), m (multiplet), br (broad). Coupling constants (*J*) are in Hz. High-resolution mass spectrometry analyses were obtained on a Shimadzu LCMS IT - TOF spectrometer. Elementary analyses were performed on a PerkinElmer CHN 2400 analyzer. Melting points were determined on a Büchi Melting Point M - 560 in a capillary tube and are uncorrected. Mass spectra were recorded with a GC–MS spectrometer working in electron ionization (EI) mode. Chiral HPLC analysis was performed on a Shimadzu HPLC using Chiralcel columns. Optical rotations were measured on a PerkinElmer 341LC spectrometer using a 1 mL cell with a 10 mm path length and are reported as follows: [α]_D_^20^ (*c* g/100 mL, solvent). Thin layer chromatography (TLC) was performed with precoated silica gel plates and visualized by potassium permanganate (KMnO_4_) staining or exposing to iodine vapor. The reaction mixtures were purified by column chromatography over silica gel (60–240 mesh). The chiral amines **7**–**12**,^21^**15**–**16**,^22^**17**,^23^**18**,^24^**25**,^25^**26**,^26^**27**,^27^ and **28**–**30**^28^ were prepared according to the literature procedures. Analytical data for those amines are in accordance with those previously reported. The reagents IBX^29^ and 4,4-dibromo-2,6-di-*tert*-butyl-cyclohexa-2,5-dienone (**20**)^30^ were synthesized according to reported procedures, and their properties matched those previously reported.

### Synthesis of Substrates (**1a**, **1b**, and **1c**)

1-Phenylphosphinan-4-one 1-oxide (**1a**), 1-borane (**1b**), and 1-sulfide (**1c**) were prepared from the free phosphine (1-phenylphosphinan-4-one) according to the modified literature procedure.^31^ A dry, argon-flushed Schlenk-flask, equipped with a magnetic stirrer and a septum, was charged with 1-phenylphosphinan-4-one (1.92 g, 0.01 mol) and dry solvent (15 mL). The solution was cooled to 0 °C when hydrogen peroxide (0.012 mol), borane-tetrahydrofuran (0.013 mol), or elemental sulfur (0.0105 mol) was slowly added to it. After 45 min at 0 °C, the solution was allowed to warm to room temperature and stirred for 24 h. The solution was evaporated, and the residue was recrystallized from Et_2_O or Et_2_O/hexane (1:2). The physical and spectral data for 1-phenylphosphinan-4-one 1-oxide (**1a**) and 1-phenylphosphinan-4-one 1-sulfide (**1c**) are in accordance with those previously reported.^31,32^ Analytical data for 1-phenylphosphinan-4-one 1-borane (1b) are described below.

### General Experimental Procedure for the Desymmetrization of 1-Phenylphosphinan-4-ones by Enantioselective Enolization^14^

The synthesis of silyl enol ethers **2b** and **2c** (the external quench procedure (EQ)) is as follows. In a flame-dried Schlenk tube (20 mL) equipped with a magnetic stirrer and inert gas inlet, the lithium amide base was formed by addition of *n*-BuLi (0.19 mL, 1.6 mol/dm^3^ solution in hexanes; 0.49 mL, 0.15 mmol) to a solution of the chiral secondary amine (0.3 mmol) and LiCl (2.1 mg, 0.05 mmol) in THF (4 mL) under nitrogen at −78 °C (dry ice/acetone bath). After 5 min, the solution was allowed to warm to room temperature and then recooled to −90 °C (methanol/liquid nitrogen bath) before addition of a solution of 1-phenylphosphinan-4-one 1-sulfide (**1c**) or 1-phenylphosphinan-4-one 1-borane (**1b**) (0.1 mmol) in THF (1 mL). After 30 min, Me_3_SiCl (0.063 mL, 0.5 mmol) was added to the reaction mixture, which was then stirred at −90 °C for further 45 min. After that time, the solution was allowed to warm to room temperature and the solvent was evaporated. The residue was quickly purified on a silica gel column (hexane/THF = 6:1) to give silyl enol ether **2b** or **2c** as a colorless oils. **2b** and **2c** are highly susceptible to hydrolysis under extraction and column chromatography conditions, and the reported yields and enantiomeric excesses refer to those determined for crude products. Enantiomeric excess of **2b** and **2c** was determined by HPLC analysis on a Chiralcel AS-H column using hexane/*i*-PrOH (90/10).

### General Procedure for the Organocatalytic α-Halogenation of 1-Phenylphosphinan-4-ones

In a flame-dried Schlenk tube (10 mL) equipped with a magnetic stirrer, the halogenating agent ((NBS, **20**, NCS, or PhICl_2_) (0.15 mmol)) was added to a mixture of phosphinanone **1a** or **1c** (21 or 22 mg, respectively, 0.1 mmol), PhCOOH (2.4 mg, 0.02 mmol), and organocatalyst (0.02 mmol) in DCM (2 mL) at 0 °C (ice/water bath), and the reaction mixture was allowed to warm to room temperature and stirred for further 16 or 24 h at that temperature. Then, in chlorination reactions, DBU (22.8 mg, 0.15 mmol) was added to effect elimination of HCl from the intermediate chloro ketone **3-Cl**, and the reaction mixture was stirred at room temperature for 1 h. In bromination reactions, the reaction mixture was warmed up to 60 °C (heating mantle) for 30 min to complete quantitative elimination of HBr from the intermediate bromo ketone **3**. Then, evaporation of the reaction mixture gave crude enone **4a** or **4c**. The crude products could be purified on a silica gel column using either DCM/THF = 10:1 for enone **4a** or hexane/THF = 8:1 for enone **4c** to give the pure products as colorless oils. Yields of **4a** and **4c** were determined by GC–MS analysis and confirmed by ^31^P NMR spectroscopy. Enantiomeric excess was determined by HPLC analysis using CSP.

### One-Pot Procedure for Direct Synthesis of Phosphin-2-en-4-one **4c** from Phosphinanone **1c**

In a flame-dried Schlenk tube (400 mL) equipped with a magnetic stirrer and inert gas inlet, the lithium amide base was formed by addition of *n*-BuLi (1.6 mol/dm^3^ solution in hexanes; 4.6 mL, 7.37 mmol) to a solution of (−)-bis[(*S*)-1-phenylethyl]amine (*S*,*S*-**5**) (3.38 mL, 14.73 mmol, 3 equiv.) and LiCl (104 mg, 2.46 mmol, 0.5 equiv.) in THF (200 mL) under nitrogen at −78 °C (dry ice/acetone bath). After 5 min, the solution was allowed to warm to room temperature and then recooled to −90 °C before addition of a solution of **1c** (1.1 g, 5 mmol) in THF (20 mL). After 30 min, Me_3_SiCl (3.1 mL, 24.5 mmol, 5 equiv.) was added to the reaction mixture, which was then stirred at −90 °C (methanol/liquid nitrogen bath) for further 45 min. After this time, the solution was allowed to warm to room temperature and the solvent was evaporated (during the evaporation, the temperature of the solution should be kept below 25 °C) to give crude silyl enol ether **2c**. The silyl enol ether **2c** was obtained in 82% yield (determined by ^31^P NMR spectroscopy) and with an enantiomeric excess of 76% (determined by chiral HPLC analysis using a Chiralcel AS-H column). Then, following the published oxidation protocol,^18^ equimolar amounts of IBX and MPO (2.06 g of IBX and 0.92 g of MPO, 1.5 equiv.) dissolved in DMSO (5 mL) were added in one portion at room temperature to the crude vacuum-dried silyl enol ether **1c** dissolved in 3 mL of DMSO. The solution was stirred vigorously for 2 h at room temperature. After this time, the reaction mixture was diluted with aqueous HCl (5%) and extracted with DCM (five times). The combined organic phase was dried (MgSO_4_), and the solvent was removed in vacuum to afford the crude product, which was further purified by silica gel column chromatography (hexane/THF = 6:1) to give enone **4c** as a light yellow oil in 48% overall yield (two steps) (0.52 g, 2.4 mmol) and with 73% ee (determined by HPLC analysis using a Chiralcel OJ-H column). Repeated recrystallizations (three times) of (−)-**4c** (73% ee) from a hexane/*i*-PrOH mixture allowed to increase its enantiopurity of the levorotatory enantiomer of **4c** left in the mother liquor up to 96% ee.

### Catalytic Desymmetrizing Dehydrogenation of Phenylphosphin-2-en-4-ones through Enamine Oxidation

Reactions were performed according to the literature procedure^20^ at room temperature. To a 10 mL flask were added phenylphosphinan-4-one **1a**–**c** (0.041–0.045 g, 0.2 mmol), catalyst (20 mol %, 0.04 mmol), pentanedioic acid (7.3 mg, 30 mol %, 0.06 mmol), and diethyl ether (0.1 mL). The reaction system was gently stirred for half an hour. Then IBX (56 mg, 0.2 mmol) was added followed by 0.1 mL of diethyl ether. After 48 h, the reaction system was diluted with ether and immediately passed through a thin layer of silica gel. The remaining organic phase was concentrated in vacuum. Yield was determined by ^31^P NMR analysis, and enantiomeric excess was determined by CSP-HPLC analysis.

#### 1-Phenylphosphinan-4-one 1-Oxide (**1a**)

This compound was prepared according to the general procedure from 1-phenylphosphinan-4-one (1.92 g, 0.01 mol) and hydrogen peroxide (30% solution in water, 1.36 mL, 0.012 mol), in acetone (15 mL). The reaction gave the corresponding oxide as the crystalline adduct **1a**_4_·(H_2_O_2_)_3_; Anal. Found: C, 56.8; H, 6.61. The adduct was practically insoluble in common organic solvents such as THF, DCM, and acetone. The formation of this type of adduct of phosphine oxides was previously reported.^33^ To decompose the adduct and remove H_2_O_2_ from **1a**, the formed crystals were melted under vacuum and heated at 180 °C (heating mantle) for 30 min to give 1.85 g (89%) of pure **1a** as white crystals, mp = 164.8–166.0 °C (lit. 164–165 °C).^34^*R_f_* = 0.16 (DCM/THF = 6:1). ^1^H NMR (500 MHz, CDCl_3_): δ 7.83–7.76 (m, 2H), 7.64–7.59 (m, 1 H), 7.58–7.52 (m, 2H), 3.24–3.11 (m, 2 H), 2.80–2.66 (m, 2H), 2.46–2.31 (m, 4H). ^13^C{^1^H} NMR (126 MHz, CDCl_3_): δ 207.6 (d, *J* = 8.2 Hz, C=O), 132.6 (d, *J* = 2.7 Hz, C_para_), 131.1 (d, *J* = 99.0 Hz, C_ipso_), 130.1 (d, *J* = 9.1 Hz, C_ortho_), 129.1 (d, *J* = 11.8 Hz, C_meta_), 36.4 (d, *J* = 6.4 Hz, C3,5), 27.2 (d, *J* = 66.0 Hz, C2,6). ^31^P{^1^H} NMR (202 MHz, CDCl_3_): δ 28.9 ppm. GC–MS (EI, 70 eV) *m*/*z* = 208.0 (10), 181.0 (10), 180.0 (100), 152.0 (46), 151.0 (13), 134.0 (29), 125.0 (80), 124.0 (86), 105.1, (37), 96.0 (13), 91.1 (12). Anal. Calcd for C_11_H_13_O_2_P: C, 64.13; H, 7.83. Found: C, 64.09; H, 7.88.

#### 1-Phenylphosphinan-4-one 1-Borane (**1b**)

This compound was prepared according to the general procedure from 1-phenylphosphinan-4-one (1.92 g, 0.01 mol) and H_3_B·THF (1.0 M solution in THF, 13 mL, 0.013 mol, 1.3 equiv.) in THF (15 mL) at room temperature for 5 h. Then, after evaporation of solvent, the product was recrystallized from hexane/Et_2_O to yield 1.69 g (82%) of **1b** as colorless crystals; mp = 94.1–96.9 °C; *R_f_* = 0.3 (hexane/THF = 8:1). ^1^H NMR (500 MHz, CDCl_3_): δ 7.82–7.75 (m, 2 H), 7.61–7.51 (m, 3H), 3.03–2.93 (m, 2H), 2.79–2.67 (m, 2H), 2.50–2.38 (m, 2H), 2.37–2.28 (m, 2H), 1.25–0.50 (bm, 3H). ^13^C{^1^H} NMR (126 MHz, CDCl_3_): δ 207.3 (d, *J* = 6.4 Hz, C=O), 132.0 (d, *J* = 2.7 Hz, C_para_), 131.2 (d, *J* = 9.1 Hz, C_ortho_), 129.3 (d, *J* = 10.0 Hz, C_meta_), 127.8 (d, *J* = 53.6 Hz, C_ipso_), 36.8 (d, *J* = 4.5 Hz, C3,5), 22.3 (d, *J* = 34.5 Hz, C2,6). ^31^P{^1^H} NMR (202 MHz, CDCl_3_): δ 3.2–1.9 (m) ppm. GC–MS (EI, 70 eV) *m*/*z* = 192.05 (64), 191.05 (31), 136.05 (18), 125.05 (21), 109.05 (21), 108.05 (100), 107.05 (52), 91.10 (19). Anal. Calcd for C_11_H_16_BOP: C, 64.13; H, 7.83. Found: C, 64.10; H, 7.85.

#### 1-Phenylphosphinan-4-one 1-Sulfide (**1c**)

This compound was prepared according to the general procedure from 1-phenylphosphinan-4-one (1.92 g, 0.01 mol) and elemental sulfur (0.335 g, 0.0105 mol, 1.05 equiv.), in toluene (15 mL). Then, after evaporation of solvent, the product was recrystallized from Et_2_O to yield 2.04 g (91%) of **1c** as white crystals; mp = 142.3–145.7 °C (lit. 144–145 °C);^34^*R_f_* = 0.26 (hexane/THF = 6:1); ^1^H NMR (500 MHz, CDCl_3_): δ 8.00–7.92 (m, 2H), 7.64–7.54 (m, 3H), 3.41–3.26 (m, 2H), 2.82–2.67 (m, 4H), 2.42–2.27 (m, 2H). ^13^C{^1^H} NMR (126 MHz, CDCl_3_): δ 207.0 (d, *J* = 7.3 Hz, C=O), 132.4 (d, *J* = 2.7 Hz, C_para_), 130.6 (d, *J* = 10.9 Hz, C_ortho_), 130.5 (d, *J* = 80.2 Hz, C_ipso_), 129.1 (d, *J* = 11.8 Hz, C_meta_), 36.8 (d, *J* = 5.5 Hz, C3,5), 31.2 (d, *J* = 50.9 Hz, C2,6). ^31^P{^1^H} NMR (202 MHz, CDCl_3_): δ 32.0 ppm. GC–MS (EI, 70 eV) *m*/*z* = 224.05 (100), 225.05 (13), 196.00 (27), 191.10 (24), 168.05 (35), 157.05 (12), 141.05 (13), 140.05 (40), 135.05 (13), 133.05 (17), 125.05 (15), 113.05 (20), 109.10 (18), 108.05 (28), 107.05 (46), 105.10 (45), 91.10 (23), 84.10 (12), 83.10 (41). Anal. Calcd for C_11_H_13_OPS: C, 58.91; H, 5.84. Found: C, 58.86; H, 5.85.

#### 1-Phenyl-4-[(trimethylsilyl)oxy]-1,2,3,6-tetrahydrophosphinine 1-Borane (**2b**)

This compound was prepared according to the general enantioselective enolization procedure from **1b** (21 mg, 0.1 mmol) to give 5 mg (22%) of **2b** as a colorless oil. Due to its very high susceptibility to hydrolysis under column chromatography conditions, borane **2b** was analyzed and used for oxidation studies only as crude; *R_f_* = 0.72 (hexane/THF = 8:1); ^1^H NMR (500 MHz, CDCl_3_): δ 7.77–7.72 (m, 2H), 7.55–7.45 (m, 3H), 5.08 (dt, *J* = 4.4 Hz and 18.6 Hz, 1H), 2.7–2.65 (m, 2H), 2.45–2.35 (m, 1H), 2.20–2.05 (m, 3H), 1.1–0.4 (bm, 3H), 0.21 (t, *J* = 3.3 Hz, 9H). ^13^C{^1^H} NMR (126 MHz, CDCl_3_): δ 151.5 (d, *J* = 12.7 Hz, C4), 131.3 (d, *J* = 11.8 Hz, C_meta_), 131.3 (d, *J* = 6.4 Hz, C_para_), 128.9 (d, *J* = 10 Hz, C_ortho_), 128.8 (d, *J* = 51.8 Hz, C_ipso_), 99.1 (d, *J* = 8.2 Hz, C5), 26.0 (d, *J* = 6.4 Hz, C3), 20.3 (d, *J* = 34.5 Hz, C6), 19.7 (d, *J* = 36.4 Hz, C2), 0.3 (C-Si). ^31^P{^1^H} NMR (202 MHz, CDCl_3_): δ −3.7 ppm. GC–MS (EI, 70 eV) *m*/*z* = 265.10 (22), 264.10 (100), 263.10 (45), 249.10 (30), 236.05 (48), 221.05 (11), 190.10 (45), 173.05 (44), 155.10 (35), 141.10 (15), 137.05 (11), 135.10 (17), 133.05 (10), 128.10 (12), 127.05 (75), 121.10 (12), 109.05 (23), 107.05 (17), 91.10 (14), 85.10 (28).

#### 1-Phenyl-4-[(trimethylsilyl)oxy]-1,2,3,6-tetrahydrophosphinine 1-Sulfide (**2c**)

This compound was prepared according to the general enantioselective enolization procedure from **1c** (22 mg, 0.1 mmol) to give 8 mg (31%) of **2c** as a colorless oil. Due to its very high susceptibility to hydrolysis under column chromatography conditions, sulfide **2c** was analyzed and used for oxidation studies only as crude; *R_f_* = 0.63 (hexane/THF = 6:1); ^1^H NMR (500 MHz, CDCl_3_): δ 7.93–7.88 (m, 2H), 7.55–7.48 (m, 3 H), 5.01 (dt, *J* = 4.50 and 26.00 Hz, 1H), 3.11–3.00 (m, 1H), 2.88–2.77 (m, 1H), 2.66–2.53 (m, 1H), 2.47–2.35 (m, 1H), 2.33–2.15 (m, 2H), 0.20 (s, 9H). ^13^C{^1^H} NMR (126 MHz, CDCl_3_): δ 151.4 (d, *J* = 15.4 Hz, C4), 132.0 (d, *J* = 78.1 Hz, C_ipso_), 131.7 (d, *J* = 3.6 Hz, C_para_), 130.4 (d, *J* = 10.0 Hz, C_ortho_), 128.8 (d, *J* = 11.8 Hz, C_meta_), 99.3 (d, *J* = 8.2 Hz, C5), 29.8 (d, *J* = 54.5 Hz, C6), 29.1 (d, *J* = 51.8 Hz, C2), 27.7 (d, *J* = 7.3 Hz, C3), 0.3 (C-Si). ^31^P{^1^H} NMR (202 MHz, CDCl_3_): δ 27.2 ppm. GC–MS (EI, 70 eV) *m*/*z* = 296.90 (22), 295.90 (55), 262.90 (16), 156.00 (28), 155.00 (100), 91.00 (13).

#### 1-Phenylphosphin-2-en-4-one 1-Oxide (*rac-***4a**)

This compound was prepared according to the general organocatalytic α-halogenation procedure from **1a** (0.57 g, 3 mmol) to give 0.36 g (64%) of *rac-***4a** as colorless crystals; mp = 106.7–107.3 °C, *R_f_* = 0.33 (CHCl_3_/THF = 10:1). ^1^H NMR (500 MHz, CDCl_3_): δ 7.79–7.74 (m, 2H), 7.65–7.60 (m, 1H), 7.58–7.48 (m, 2H), 7.10–7.04 (m, 1H), 6.73 (dd, *J* = 13.6 and 23.0 Hz, 1H), 3.15 (s, 1H), 2.85–2.73 (m, 1H), 2.62–2.51 (m, 1H), 2.49–2.40 (m, 1H). ^13^C{^1^H} NMR (126 MHz, CDCl_3_): δ 196.1 (d, *J* = 14.5 Hz, C=O), 142.6 (C3), 138.2 (d, *J* = 83.6 Hz, C2), 132.9 (d, *J* = 2.7 Hz, C_para_), 130.6 (d, *J* = 10.9 Hz, C_ortho_), 129.9 (d, *J* = 105.4 Hz, C_ipso_), 129.2 (d, *J* = 12.7 Hz, C_meta_), 33.8 (d, *J* = 6.4 Hz, C5), 26.5 (d, *J* = 70.8 Hz, C6). ^31^P{^1^H} NMR (202 MHz, CDCl_3_): δ 16.9 ppm. GC–MS (EI, 70 eV) *m*/*z* = 178.00 (33), 150.00 (19), 132.05 (10), 131.05 (100), 124.00 (24), 103.05 (14). Anal. Calcd for C_11_H_11_O_2_P: C, 64.08; H, 5.38. Found: C, 64.01; H, 5.24.

#### 1-Phenylphosphin-2-en-4-one 1-Oxide (−)**-4a**

This compound was prepared according to the general organocatalytic α-halogenation procedure from **1a** (0.19 g, 1 mmol) to give 0.1 g, (54%) of (−)-**4a** as colorless crystals; ([α]_D_^20^ = −152.4 (*c* 1.1, CHCl_3_) for ee = 54%); mp = 110.7–113.6 °C; *R_f_* = 0.33 (CHCl_3_/THF = 10:1). CSP-HPLC conditions: Chiralcel OD-H, hexane/2-propanol = 90:10, 1 mL/min, retention time = 27.8 min for the major enantiomer and 32.3 min for the minor enantiomer. ^1^H NMR (500 MHz, CDCl_3_): δ 7.79–7.74 (m, 2H), 7.65–7.60 (m, 1H), 7.58–7.48 (m, 2H), 7.10–7.04 (m, 1H), 6.73 (dd, *J* = 13.6 and 23.0 Hz, 1H), 3.15 (s, 1H), 2.85–2.73 (m, 1H), 2.62–2.51 (m, 1H), 2.49–2.40 (m, 1H). ^13^C{^1^H} NMR (126 MHz, CDCl_3_): δ 196.1 (d, *J* = 14.5 Hz, C=O), 142.6 (C3), 138.2 (d, *J* = 83.6 Hz, C2), 132.9 (d, *J* = 2.7 Hz, C_para_), 130.6 (d, *J* = 10.9 Hz, C_ortho_), 129.9 (d, *J* = 105.4 Hz, C_ipso_), 129.2 (d, *J* = 12.7 Hz, C_meta_), 33.8 (d, *J* = 6.4 Hz, C5), 26.6 (d, *J* = 70.8 Hz, C6). ^31^P{^1^H} NMR (202 MHz, CDCl_3_): *δ* 16.9 ppm. GC–MS (EI, 70 eV) *m*/*z* = 178.00 (33), 150.00 (19), 132.05 (10), 131.05 (100), 124.00 (24), 103.05 (14). Anal. Calcd for C_11_H_11_O_2_P: C, 64.08; H, 5.38. Found: C, 64.27; H, 5.43.

#### 1-Phenylphosphin-2-en-4-one 1-Sulfide (*rac***-4c**)

This compound was prepared according to the general one-pot procedure from **1c** (0.41 g, 2 mmol) to give 0.19 g, 0.86 mmol, 51% overall yield after two steps, as colorless crystals; mp = 91.9–92.4 °C; *R_f_* = 0.36 (hexane/THF = 6:1). ^1^H NMR (500 MHz, CDCl_3_): δ 7.96–7.89 (m, 2H), 7.66–7.61 (m, 1H), 7.61–7.55 (m, 2H), 7.05–6.95 (m, 1H), 6.63 (dd, *J* = 12.5 and 34.5 Hz, 1H), 3.30 (tdd, *J* = 4.6, 12.2 and 16.5 Hz, 1H), 2.95–2.73 (m, 2H), 2.62–2.49 (m, 1H). ^13^C{^1^H} NMR (126 MHz, CDCl_3_): δ 195.6 (d, *J* = 13.6 Hz, C=O), 139.3 (d, *J* = 3.6 Hz, C3), 139.2 (d, *J* = 68.2 Hz, C2), 132.7 (d, *J* = 3.6 Hz, C_para_), 131.1 (d, *J* = 11.8 Hz, C_ortho_), 129.6 (d, *J* = 85.7 Hz, C_ipso_), 129.1 (d, *J* = 11.8 Hz, C_meta_), 34.0 (d, *J* = 7.3 Hz, C5), 31.3 (d, *J* = 55.4 Hz, C6). ^31^P{^1^H} NMR (202 MHz, CDCl_3_): δ 21.3 ppm. GC–MS (EI, 70 eV) *m*/*z* = 223.05 (13), 222.05 (100), 190.10 (14), 189.10 (86), 171.10165.05 (10), (12), 143.15 (12), 142.15 (96), 141.15 (18), 140.05 (50), 134.10 (22), 133.10 (50), 131.10 (20), 109.10 (11), 108.10 (28), 107.10 (69), 105.15 (24), 103.10 (18), 91.10 (10), 83.05 (12), 81.05 (11). Anal. Calcd for C_11_H_11_OPS: C, 59.45; H, 4.99. Found: C, 59.39; H, 4.95.

#### 1-Phenylphosphin-2-en-4-one 1-Sulfide (−)**-4c**

The compound (*−*)-**4c** was prepared according to the general one-pot procedure from **1c** (1.1 g, 5 mmol) to give 0.52 g, 2.4 mmol, 48% overall yield after two steps; colorless oil; ([α]_D_^20^ = −86.17 (*c* 1.5, CHCl_3_) for ee = 96%); *R_f_* = 0.36 (hexane/THF = 6:1); CSP-HPLC conditions: Chiralcel OJ-H, hexane/2-propanol = 95:5, 1 mL/min, retention time = 66 min for the minor enantiomer and 70 min for the major enantiomer. ^1^H NMR (500 MHz, CDCl_3_): δ 7.96–7.89 (m, 2H), 7.66–7.61 (m, 1H), 7.61–7.55 (m, 2H), 7.05–6.95 (m, 1H), 6.63 (dd, *J* = 12.5 and 34.5 Hz, 1H), 3.30 (tdd, *J* = 4.6, 12.2 and 16.5 Hz, 1H), 2.95–2.73 (m, 2H), 2.62–2.49 (m, 1H). ^13^C{^1^H} NMR (126 MHz, CDCl_3_): δ 195.6 (d, *J* = 13.6 Hz, C=O), 139.3 (d, *J* = 3.6 Hz, C3), 139.2 (d, *J* = 68.2 Hz, C2), 132.7 (d, *J* = 3.6 Hz, C_para_), 131.1 (d, *J* = 11.8 Hz, C_ortho_), 129.6 (d, *J* = 85.7 Hz, C_ipso_), 129.1 (d, *J* = 11.8 Hz, C_meta_), 34.0 (d, *J* = 7.3 Hz, C5), 31.3 (d, *J* = 55.4 Hz, C6). ^31^P{^1^H} NMR (202 MHz, CDCl_3_): δ 21.3 ppm. GC–MS (EI, 70 eV) *m*/*z* = 223.05 (13), 222.05 (100), 190.10 (14), 189.10 (86), 171.10165.05 (10), (12), 143.15 (12), 142.15 (96), 141.15 (18), 140.05 (50), 134.10 (22), 133.10 (50), 131.10 (20), 109.10 (11), 108.10 (28), 107.10 (69), 105.15 (24), 103.10 (18), 91.10 (10), 83.05 (12), 81.05 (11). Anal. Calcd for C_11_H_11_OPS: C, 59.45; H, 4.99. Found: C, 59.15; H, 4.78.

#### 1-Phenylphosphin-2,5-dien-4-one 1-Oxide (**31a**)

This compound was prepared according to the catalytic desymmetrizing dehydrogenation procedure from 1-phenylphosphinan-4-one **1a**. **31a**: 8 mg, 0.004 mmol, (2%); pale yellow crystals; mp = 131.2–132.6 °C (lit. 130–131 °C);^35^*R_f_* = 0.45 (DCM/THF = 6:1); ^1^H NMR (500 MHz, CDCl_3_): δ 7.81–7.75 (m, 2H), 7.64 (dd, *J* = 1.7 and 7.4 Hz, 1H), 7.59–7.54 (m, 2H), 7.16–7.09 (m, 2H), 6.93–6.88 (m, 1H), 6.86–6.81 (m, 1H). ^13^C{^1^H} NMR (126 MHz, CDCl_3_): δ 183.0 (d, *J* = 25.0 Hz, C=O), 140.4 (d, *J* = 2.7 Hz, C3,5), 137.8 (d, *J* = 90.5 Hz, C2,6), 133.2 (d, *J* = 2.7 Hz, C_para_), 130.9 (d, *J* = 10.9 Hz, C_ortho_), 129.3 (d, *J* = 13.6 Hz, C_meta_), 127.7 (d, *J* = 111.5 Hz, C_ipso_). ^31^P{^1^H} NMR (202 MHz, CDCl_3_): δ −1.3 ppm. GC–MS (EI, 70 eV) *m*/*z* = 158.0 (14), 157.0 (100), 150.0 (15), 147.0 (20), 131.0 (13), 129.0 (33), 128.0 (17), 124.0 (14), 103.0 (12), 77.0 (52), 51.0 (37), 50.0 (11), 47.0 (20). Anal. Calcd for C_11_H_9_O_2_P: C, 64.71; H, 4.44. Found: C, 64.80; H, 4.59.

## Supporting Information Available

Table S1 - Complete catalyst screening of enantioselective α-bromination of **1a** and **1c**; Table S2 - Catalytic desymmetrizing dehydrogenation of **1a**–**c** through enamine oxidation; Figure S1 - CSP-HPLC traces of optically active **4c**; ^1^H NMR, ^13^C{^1^H} NMR, and ^31^P{^1^H} NMR spectra (PDF)
