# Supplementary material for: Desymmetrization Approach to the Synthesis of Optically Active P-Stereogenic Phosphin-2-en-4-ones
Source: J Org Chem. 2021 Apr 26;86(9):6195–206. doi: 10.1021/acs.joc.0c03055 (PMC8279496; doi:10.1021/acs.joc.0c03055)
Supplement: Supplementary file 1 — jo0c03055_si_001.pdf [file jo0c03055_si_001.pdf]

SUPPORTING INFORMATION

Desymmetrization approach to the synthesis of optically active P-stereogenic phosphin-2-en-4-ones

Elżbieta Łastawiecka,[a] Sławomir Frynas,[a] K. Michał Pietrusiewicz*[a]

Table of Contents

1. Table S1. Complete Catalysts Screening Data for Enantioselective α-Bromination of **1a** and **1c**. **S2**

2. Table S2. Catalytic Desymmetrizing Dehydrogenation of Phenylphosphin-2-en-4-ones **1a-c** through Enamine Oxidation **S3**

3. Figure S1. Copies of CSP-HPLC traces of optically active **4c** **S4**

4. Copies of ^{1}H NMR ^{13}C NMR and ^{31}P NMR spectra **S5**

[a] Department of Organic Chemistry, Institute of Chemical Sciences, Faculty of Chemistry, Maria Curie - Sklodowska University, Gliniana St. 33, Lublin 20 - 614, Poland.

Table S1. Complete Screening Data of Enantioselective α-Bromination of **1a** and **1c**[a]

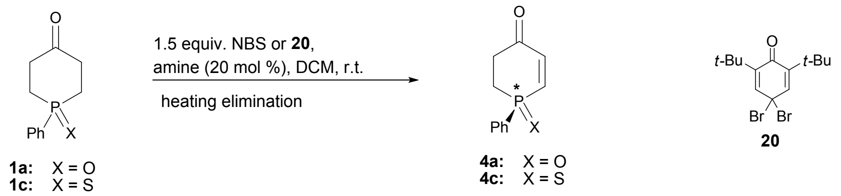

Entry	Substrate	Catalyst	Additive (mol %)	Condition	Yield of 4 [%][b]	ee [%][c]
1	**1a**	**5**	-	NBS	13	9
2	**1a**	**6**	-	NBS	8	20
3	**1a**	**7**	-	NBS	9	20
4	**1a**	**12**	-	NBS	29	5
5	**1a**	**13**	-	NBS	48	40
6	**1a**	**13**	PhCOOH (20)	NBS	54	25
7	**1a**	**14**	-	NBS	59	9
8	**1a**	**14**	PhCOOH (20)	NBS	31	26
9	**1a**	**15**	-	NBS	44	30
10	**1a**	**15**	PhCOOH (20)	NBS	23	48
11	**1a**	**16**	-	NBS	52	29
12	**1a**	**16**	PhCOOH (20)	NBS	41	23
13	**1a**	**17**	-	NBS	58	9
14	**1a**	**17**	PhCOOH (20)	NBS	32	23
15	**1a**	**(*S*)-proline (19)**	-	NBS	45	10
16	**1a**	**(*S*)-proline (19)**	PhCOOH (20)	NBS	47	34
17	**1a**	**L-hydroxyloproline**		NBS	5	75
18	**1a**	**21**	-	NBS	12	15
19	**1a**	**21**	PhCOOH (20)	NBS	35	38
20	**1a**	**22**	-	NBS	34	42
21	**1a**	**22**	PhCOOH (20)	NBS	44	33
22	**1a**	**23**	-	NBS	27	39
23	**1a**	**23**	PhCOOH (20)	NBS	34	30
24	**1a**	**24**	-	NBS	54	**55**
25	**1a**	**24**	PhCOOH (20)	NBS	26	41
26	**1a**	**25**	-	NBS	51	28
27	**1a**	**25**	PhCOOH (20)	NBS	74	27
28	**1a**	**25**	PhCOOH (20)	**20**	47	11
29	**1a**	**26**	PhCOOH (20)	NBS	3	77
30	**1a**	**26**	PhCOOH (20)	**20**	58	8
31	**1a**	**27**	PhCOOH (20)	NBS	1	n.d.
32	**1a**	**28**	PhCOOH (20)	NBS	4	n.d.
33	**1a**	**29**	PhCOOH (20)	NBS	15	4
34	**1a**	**30**	PhCOOH (20)	NBS	43	14
35	**1a**	**L-serine**	-	NBS	60	11
36	**1a**	**L-serine**	PhCOOH (20)	NBS	1	87
37[(72h)]	**1a**	**L-serine**	PhCOOH (20)	NBS	12	40
38	**1a**	**L-threonine**	-	NBS	4	47
39	**1a**	**L-alanine**	-	NBS	1	86
40[(72h)]	**1a**	**L-alanine**	-	NBS	3	70
41	**1a**	**L-leucyne**	-	NBS	3	72
42	**1a**	**L-valine**	-	NBS	2	69
43	**1c**	**5**	PhCOOH (20)	**20**	76	24
44	**1c**	**5**	-	**20**	75	15
45	**1c**	**6**	PhCOOH (20)	**20**	76	24
46	**1c**	**13**	PhCOOH (20)	**20**	68	16
47	**1c**	**14**	PhCOOH (20)	**20**	56	16

48	**1c**	**15**	PhCOOH (20)	**20**	63	8
49	**1c**	**17**	PhCOOH (20)	**20**	84	20
50	**1c**	**(S)-proline (19)**	PhCOOH (20)	**20**	57	24
51	**1c**	**L-hydroxyproline**	-	**20**	67	23
52	**1c**	**L-hydroxyproline**	PhCOOH (20)	**20**	73	19
53	**1c**	**21**	-	**20**	63	14
54	**1c**	**21**	PhCOOH (20)	**20**	70	22
55	**1c**	**22**	-	**20**	67	17
56	**1c**	**22**	PhCOOH (20)	**20**	73	20
57	**1c**	**23**	-	**20**	54	21
58	**1c**	**23**	PhCOOH (20)	**20**	70	22
59	**1c**	**24**	-	**20**	77	17
60	**1c**	**24**	PhCOOH (20)	**20**	63	4
61	**1c**	**25**	-	**20**	84	33
62	**1c**	**25**	PhCOOH (20)	**20**	87	22
63	**1c**	**26**	-	**20**	63	14
64	**1c**	**26**	PhCOOH (20)	**20**	66	38
65	**1c**	**L-serine**	-	**20**	65	17
66	**1c**	**L-serine**	PhCOOH (20)	**20**	67	23
67	**1c**	**L-threonine**	-	**20**	65	20
68	**1c**	**L-threonine**	PhCOOH (20)	**20**	67	29
69	**1c**	**L-alanine**	-	**20**	63	29
70	**1c**	**L-alanine**	PhCOOH (20)	**20**	40	27
71	**1c**	**L-valine**	-	**20**	70	15

[a] Procedure: To a mixture of **1a** or **1c** (0.1 mmol), additive and catalyst in DCM (2 mL) was added NBS or **20** (0.15 mmol), and the reaction mixture was stirred at ambient temperature for the time indicated. [b] Yields of **4a,c** determined by GC-MS analysis and confirmed by ^{31}P NMR spectroscopy. [c] Enantiomeric excess **4a** and **4c** determined by CSP-HPLC analysis using Chiralcel OD-H and O*J*-H column, respectively.

Table S2. Catalytic desymmetrizing dehydrogenation of phenylphosphinan-4-ones through enamine oxidation (**1a**-**1c**).[a]

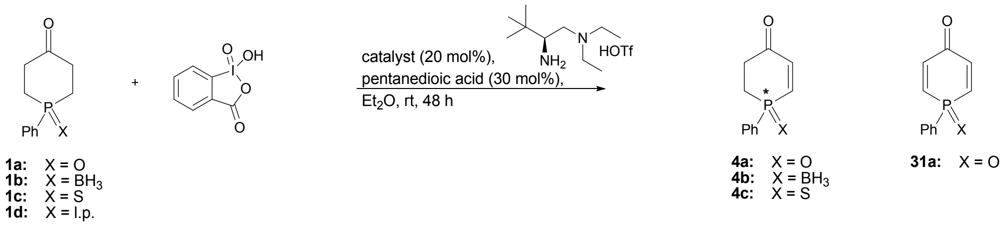

Entry	Substrate	Conversion	Yield of enone 4 a-c [%][b]	ee of enone 4 a-c [%][c]	Byproducts
1	**1a**	21%	19	21	2% of **31a**
2	**1b**	37%	0%	n.d.	12% fo **1a** (messy reaction)
3	**1c**	1%	0%	n.d.	-
4	**1d**	97%	0%	n.d.	97% of **1a**

[a] Procedure: To a mixture of **1c** (0.1 mmol), additive and catalyst in the indicated solvent (0.5 mL) was added NBS (0.15 mmol), and the reaction mixture was stirred at ambient temperature for the time indicated. [b] Yields of **4c** determined by GC-MS analysis and confirmed by ^{31}P NMR spectroscopy. [c] Enantiomeric excess determined by CSP-HPLC analysis using Chiralcel OD- H column.

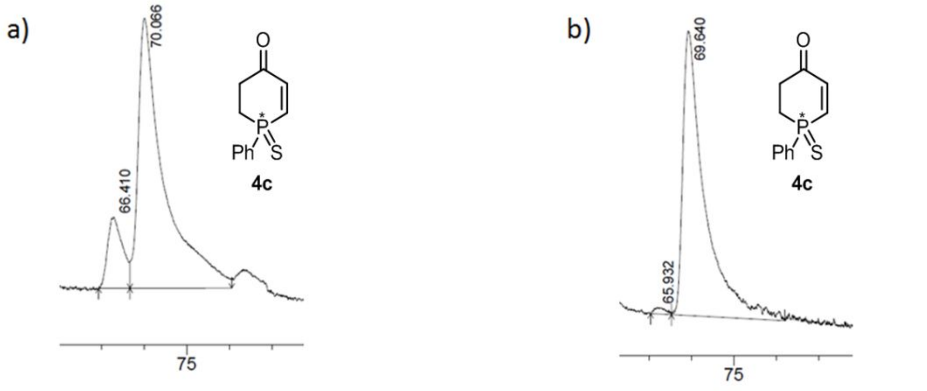

Peak#	Ret. Time	Area %
1	66.410	13.309
2	70.066	86.691
Total		100.000

Peak#	Ret. Time	Area %
1	65.932	2.256
2	69.640	97.744
Total		100.000

a) - trace of **4c** isolated from the synthesis on 1.1 g scale.

b) - trace of **4c** after three-times recrystallization of the isolated **4c** of 73% ee.

[a]HPLC conditions: CHIRALCEL O*J*-H, hexane/2-propanol = 95:5, 1 mL/min.

Figure S1. Copies of CSP-HPLC traces of optically active **4c**[a]

Copies of ^{1}H NMR ^{13}C NMR and ^{31}P NMR spectra

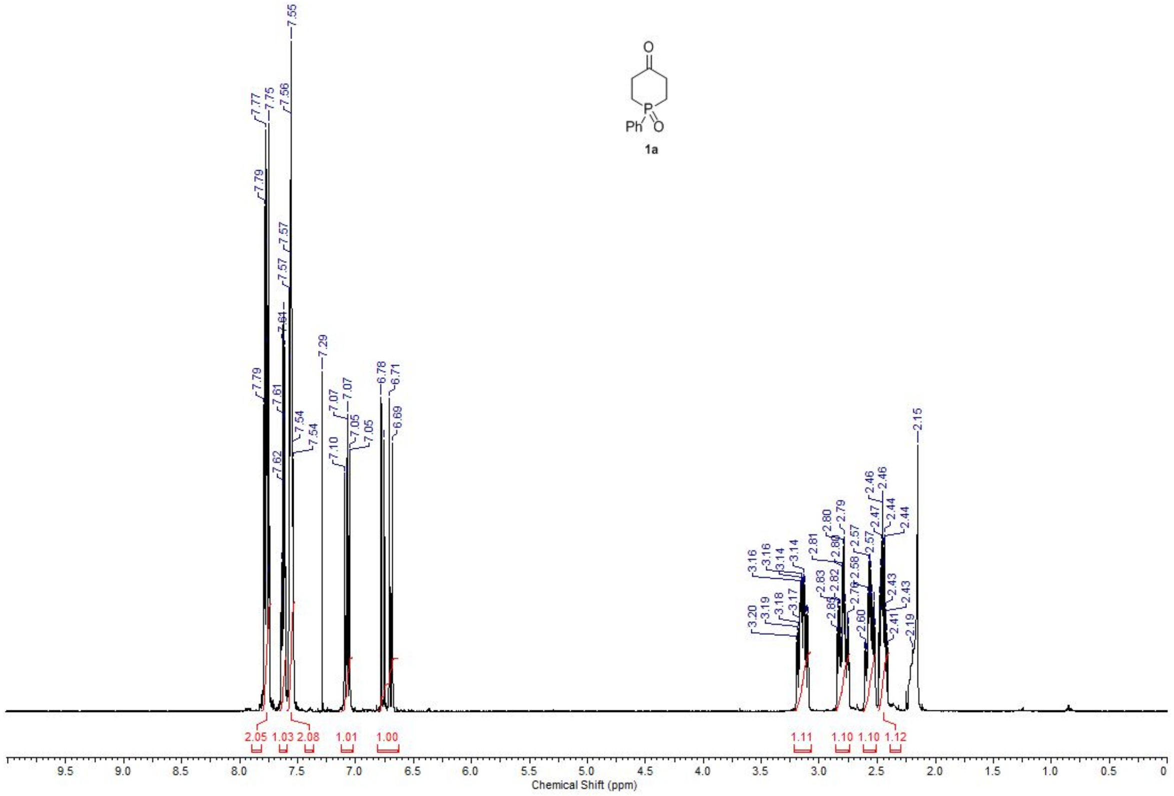

^{1}H NMR spectrum of 1-phenylphosphinan-4-one 1-oxide (**1a**) ($CDCl_3$, 500 MHz).

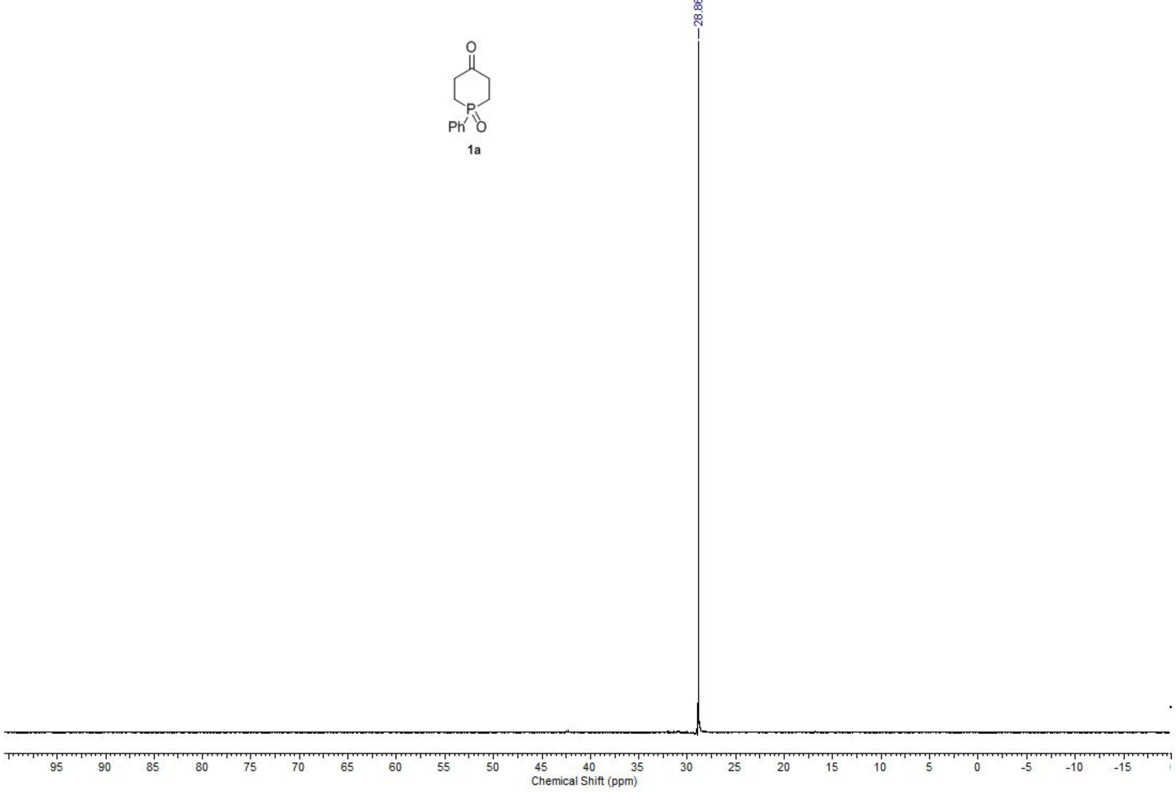

^{31}P{^{1}H} NMR spectrum of 1-phenylphosphinan-4-one 1-oxide (**1a**) ($CDCl_3$, 202 MHz).

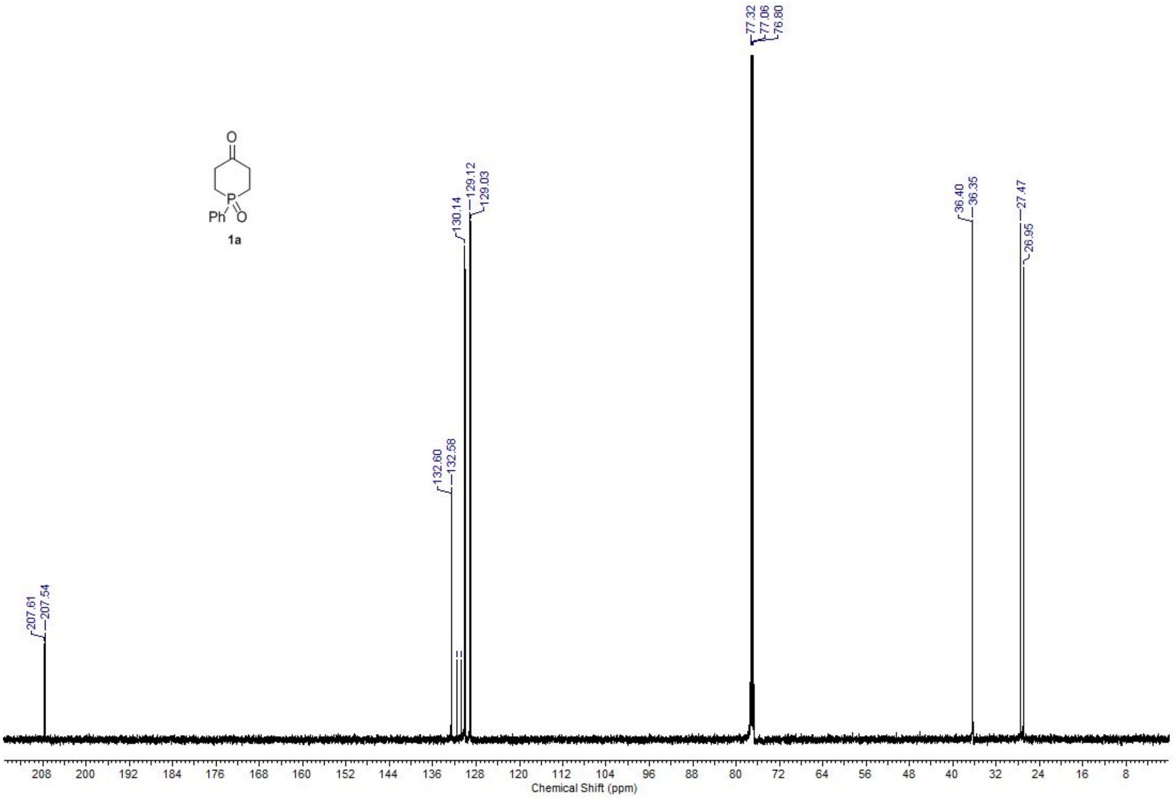

$^{31}C\{^{1}H\}$ NMR spectrum of 1-phenylphosphinan-4-one 1-oxide (**1a**) ($CDCl_3$, 126 MHz).

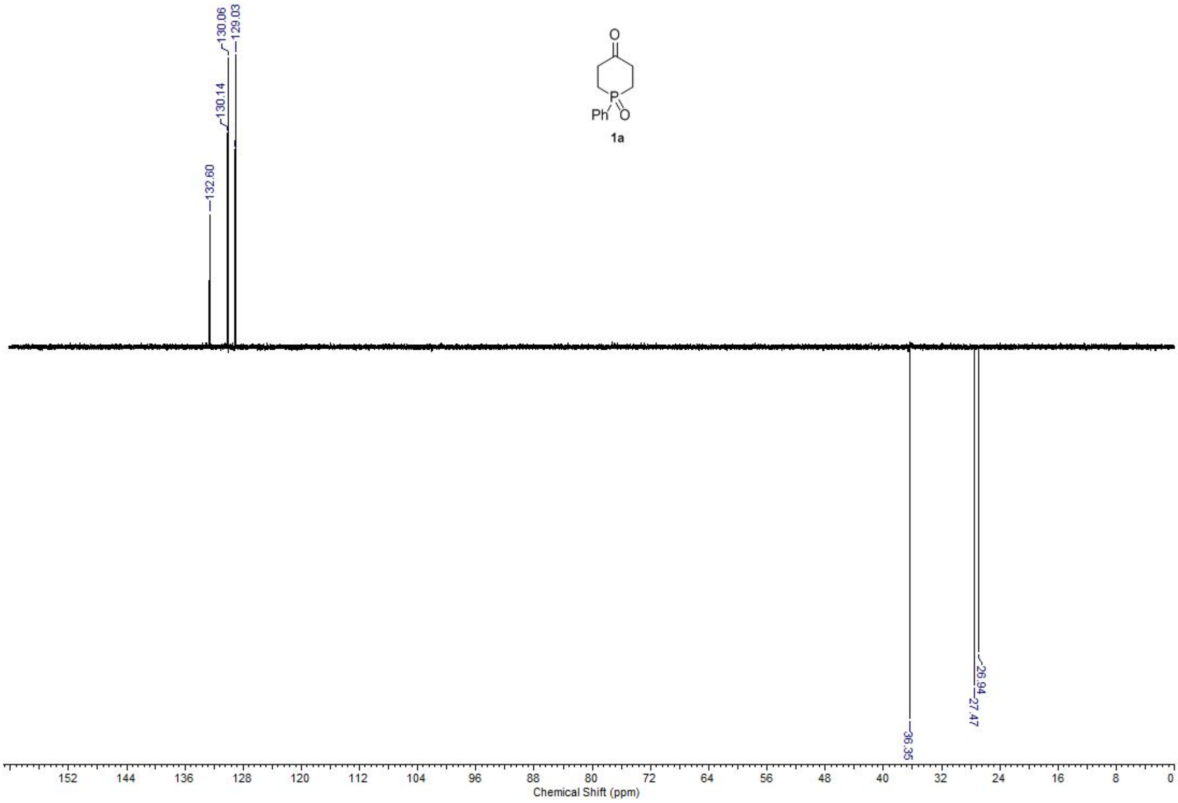

DEPT 135 spectrum of 1-phenylphosphinan-4-one 1-oxide (**1a**) ($CDCl_3$, 126 MHz).

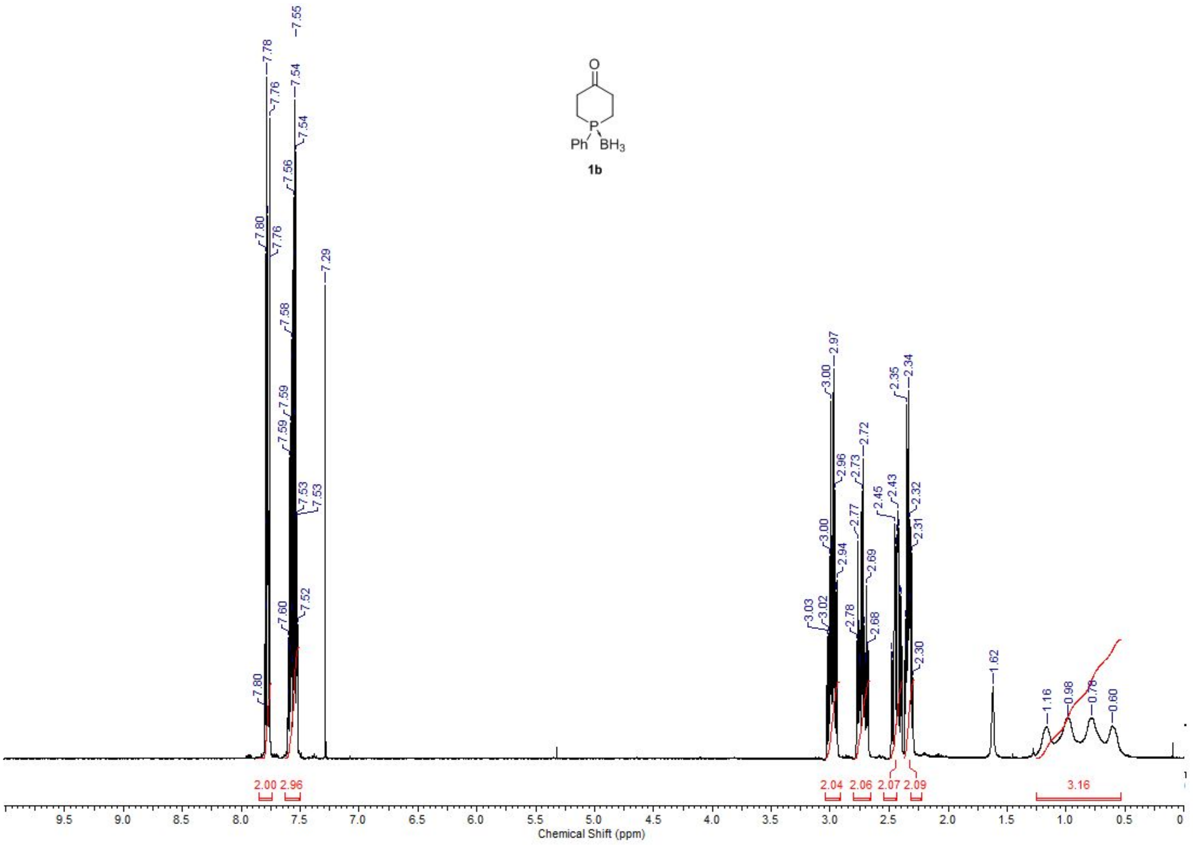

^{1}H NMR spectrum of 1-phenylphosphinan-4-one 1-borane (**1b**) ($CDCl_3$, 500 MHz).

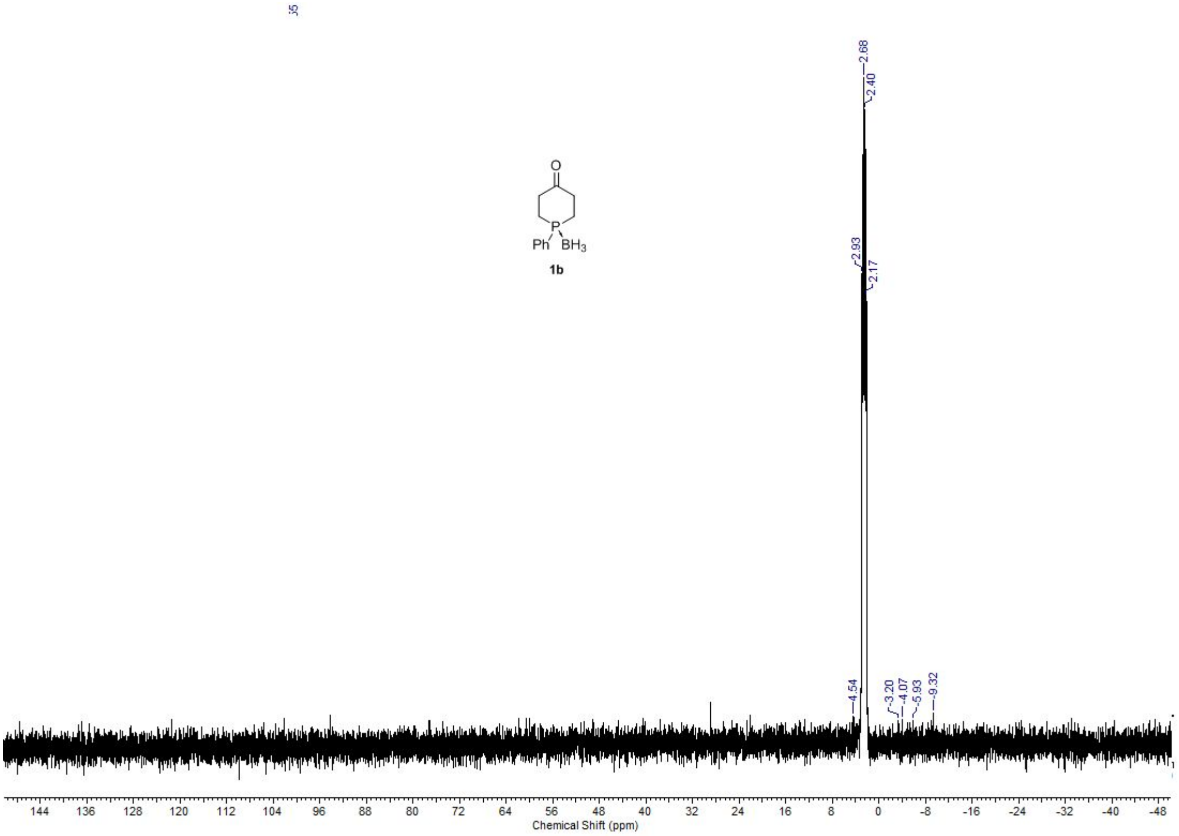

$^{31}P\{^{1}H\}$ NMR spectrum of 1-phenylphosphinan-4-one 1-borane (**1b**) ($CDCl_3$, 202 MHz).

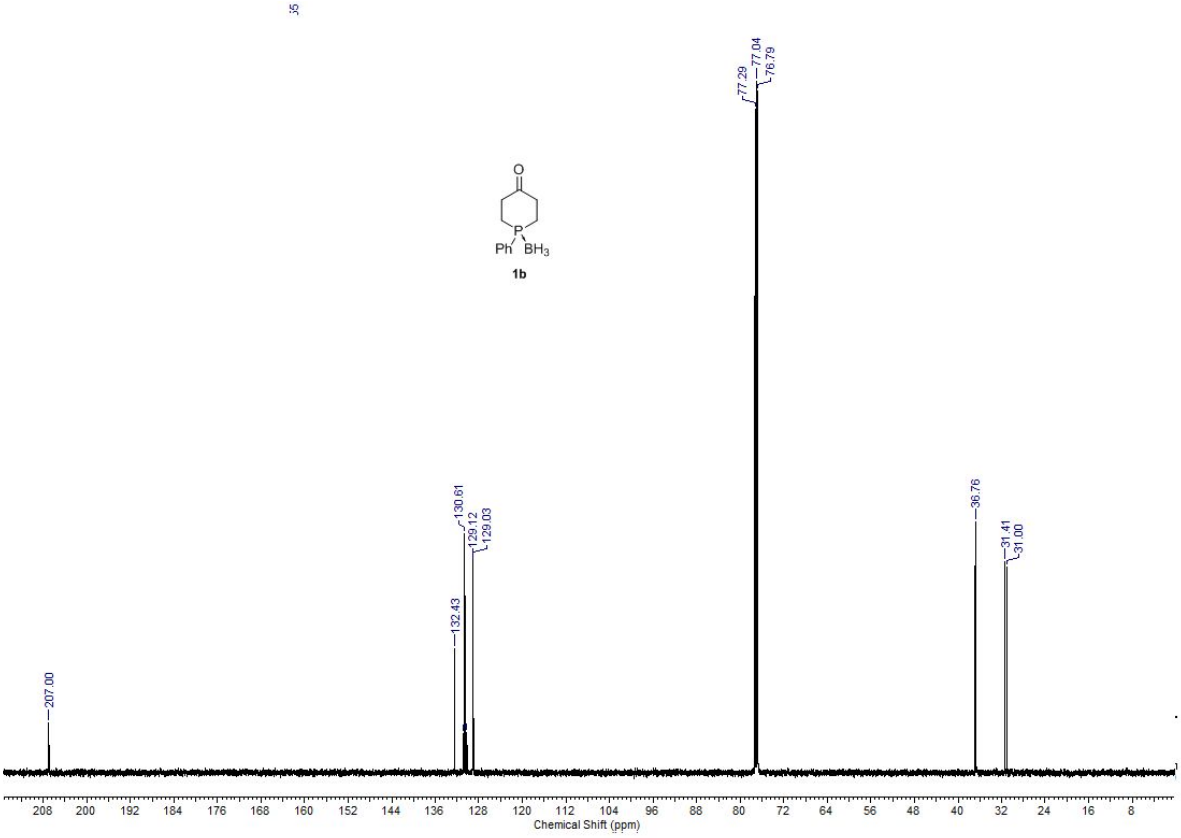

^{31}C{^{1}H} NMR spectrum of 1-phenylphosphinan-4-one 1-borane (**1b**) ($CDCl_3$, 126 MHz).

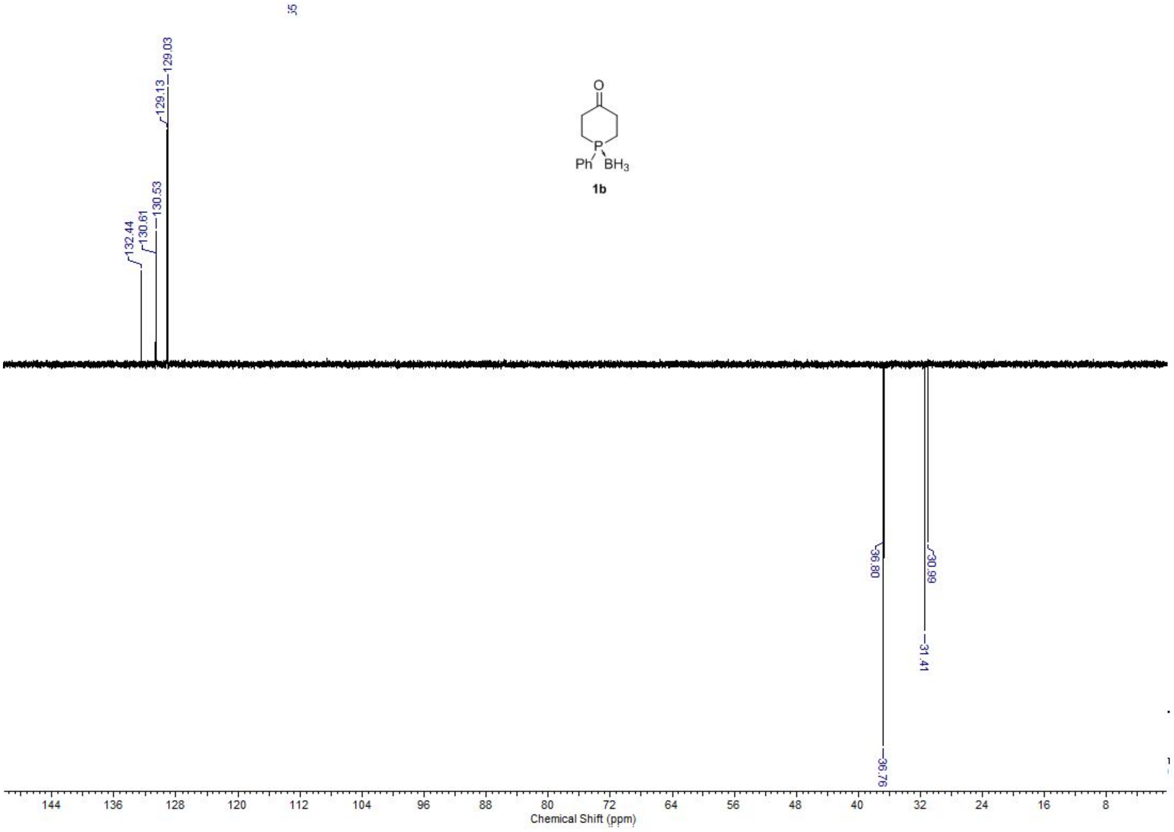

DEPT 135 spectrum of 1-phenylphosphinan-4-one 1-borane (**1b**) ($CDCl_3$, 126 MHz).

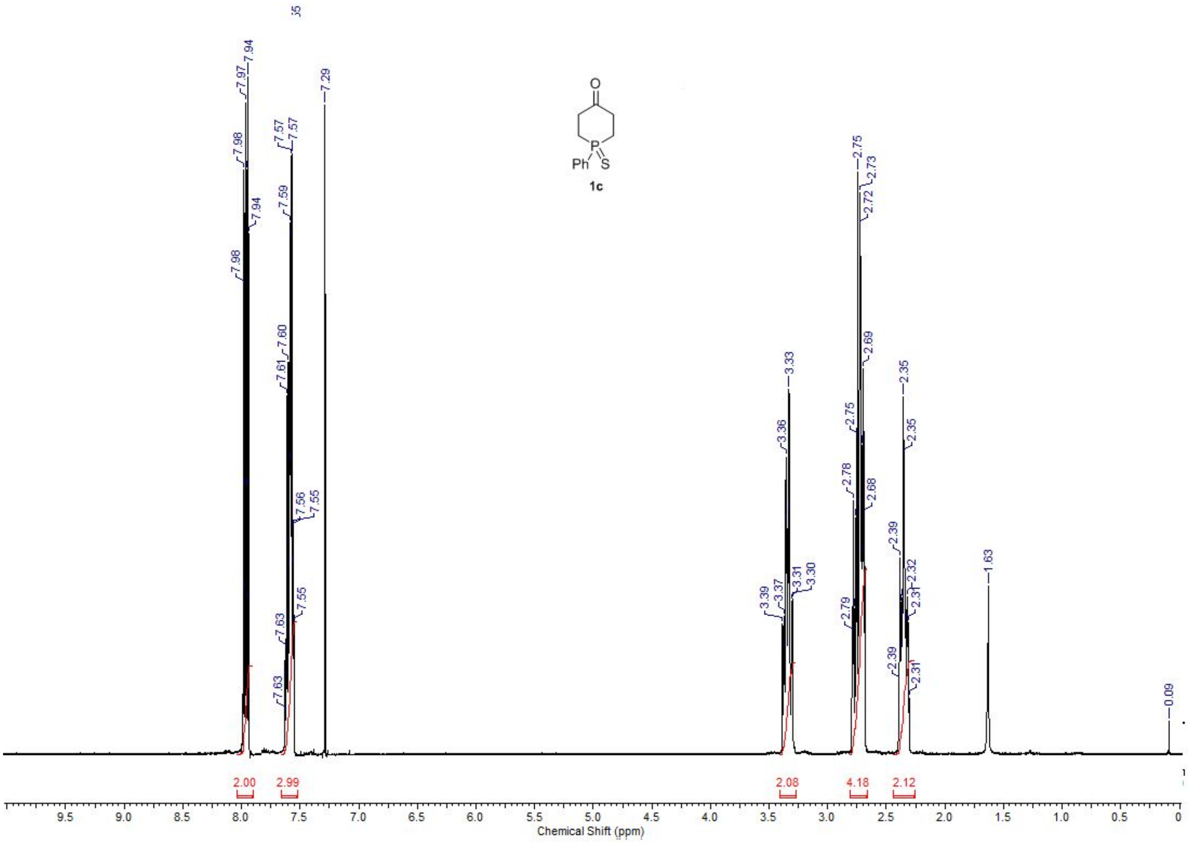

^{1}H NMR spectrum of 1-phenylphosphinan-4-one 1-sulfide (**1c**) ($CDCl_3$, 500 MHz).

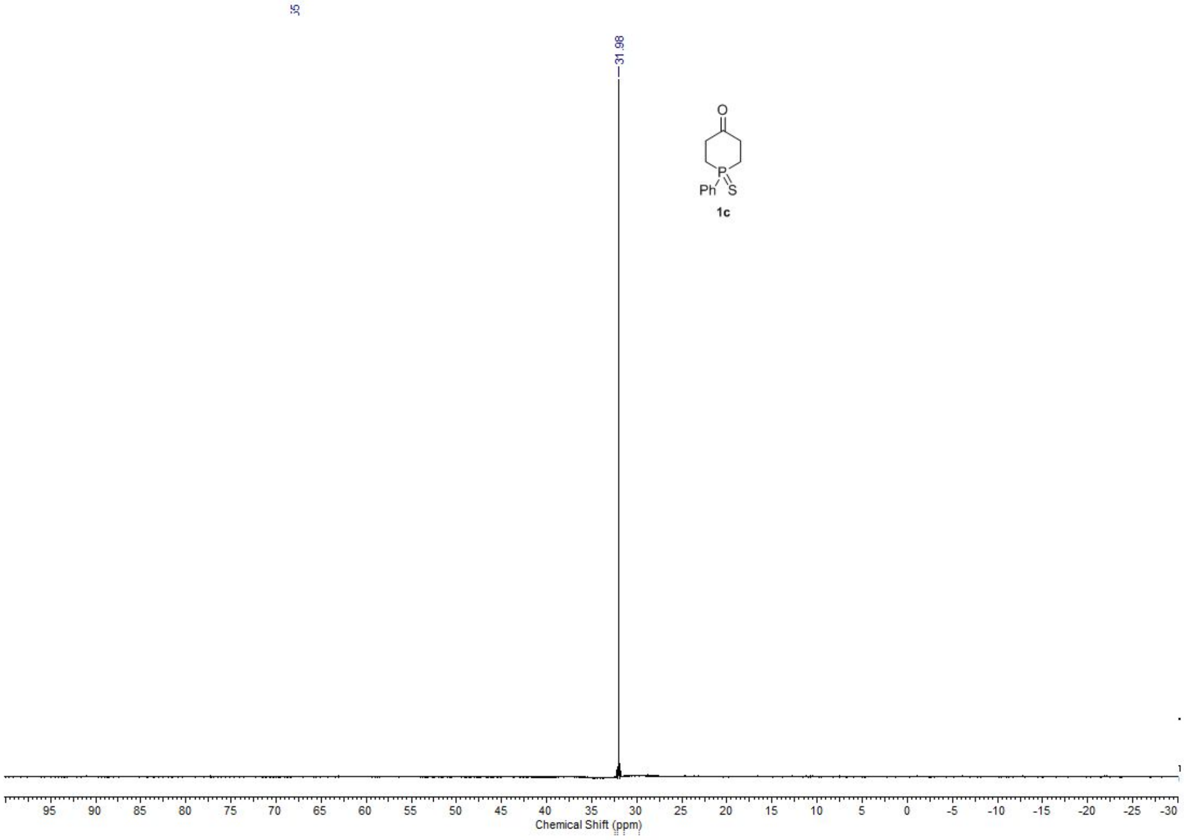

$^{31}P\{^{1}H\}$ NMR spectrum of 1-phenylphosphinan-4-one 1-sulfide (**1c**) ($CDCl_3$, 202 MHz).

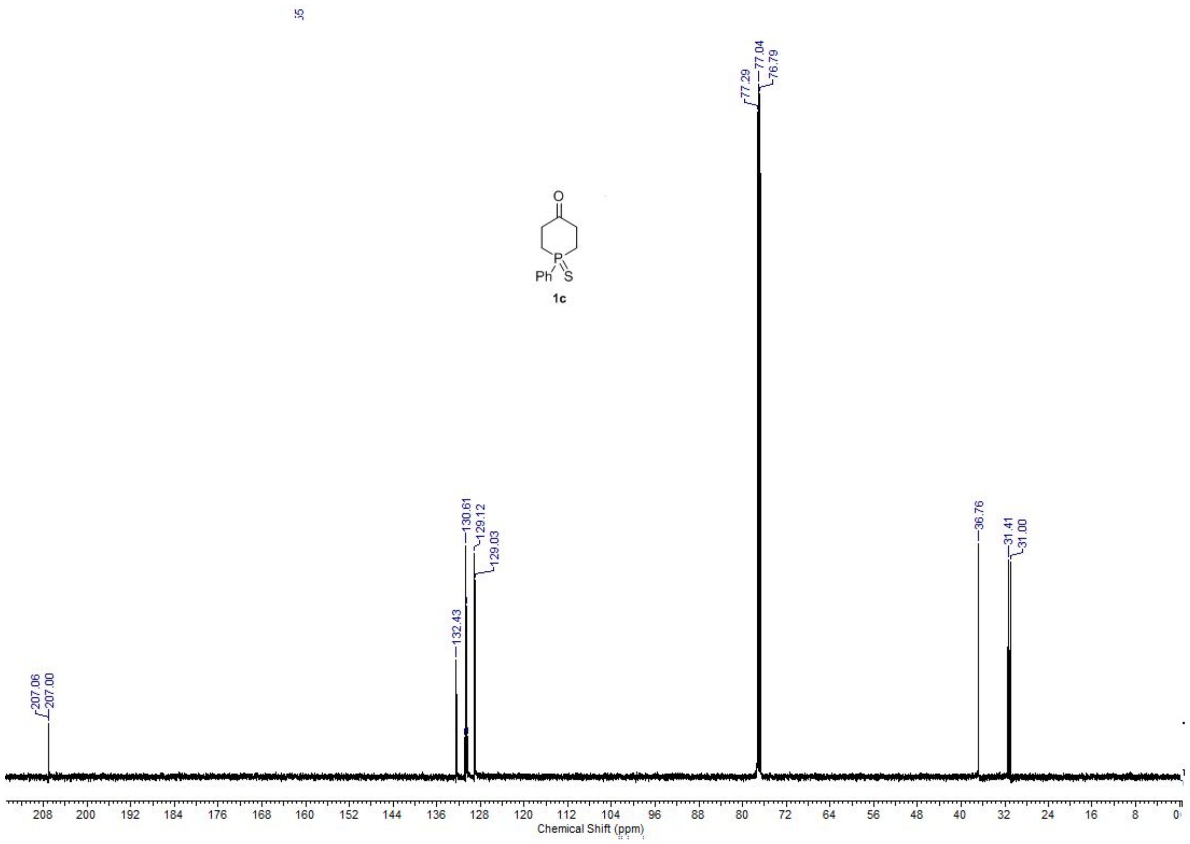

^{31}C{^{1}H} NMR spectrum of 1-phenylphosphinan-4-one 1-sulfide (**1c**) ($CDCl_3$, 126 MHz).

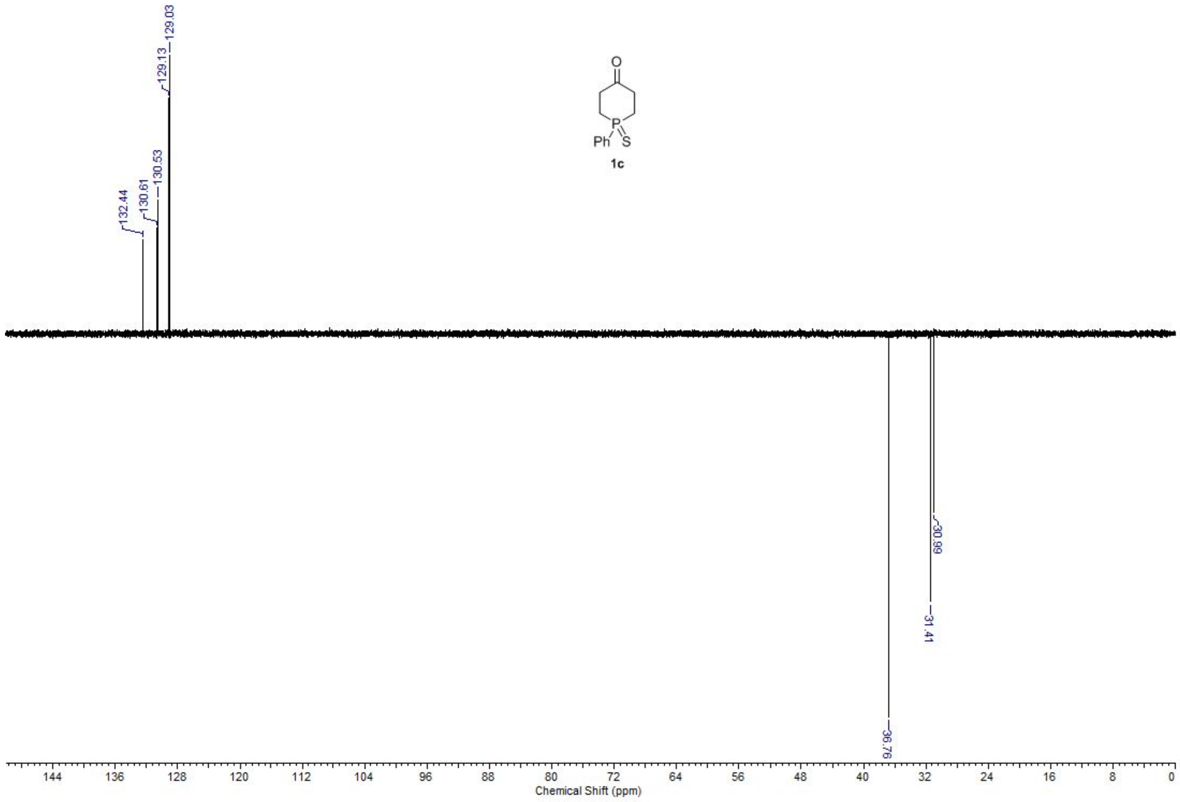

DEPT 135 spectrum of 1-phenylphosphinan-4-one 1-sulfide (**1c**) ($CDCl_3$, 126 MHz).

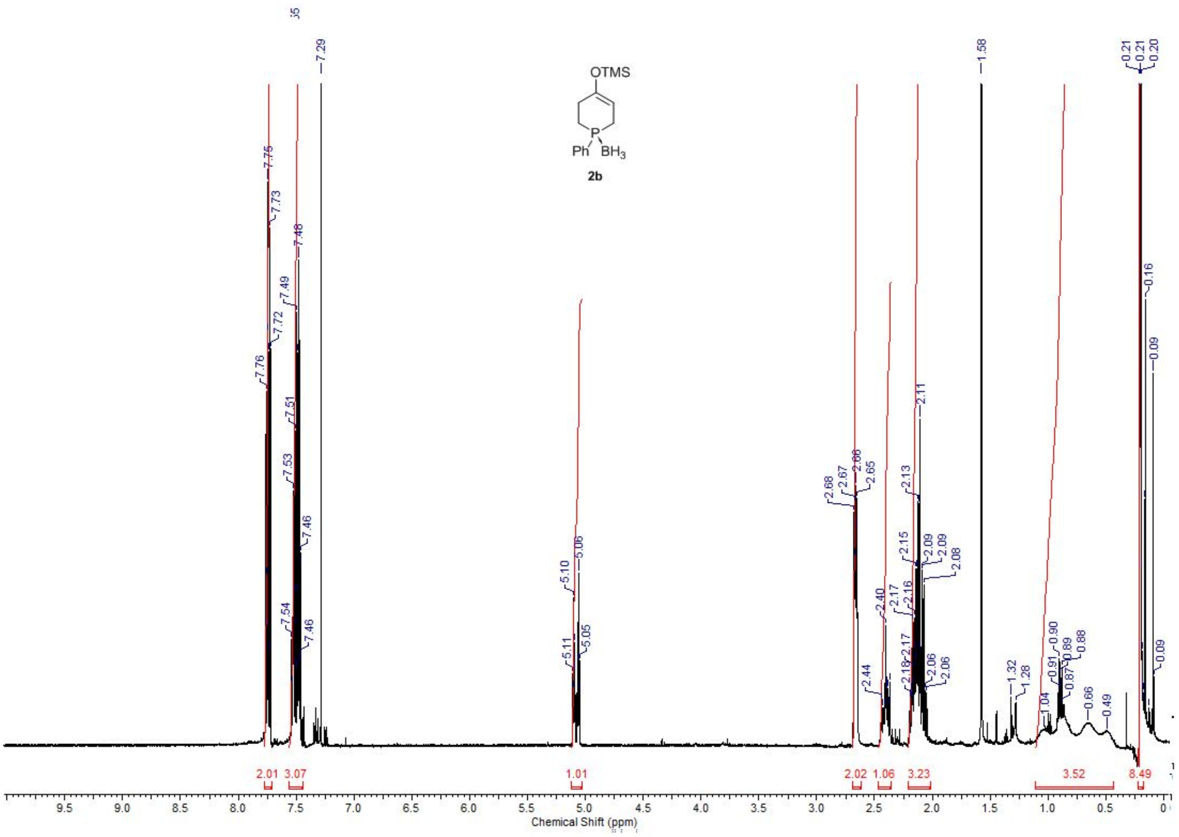

^{1}H NMR spectrum of 1-phenyl-4-[(trimethylsilyl)oxy]-1,2,3,6-tetrahydrophosphinine 1-borane (**2b**) ($CDCl_3$, 500 MHz).

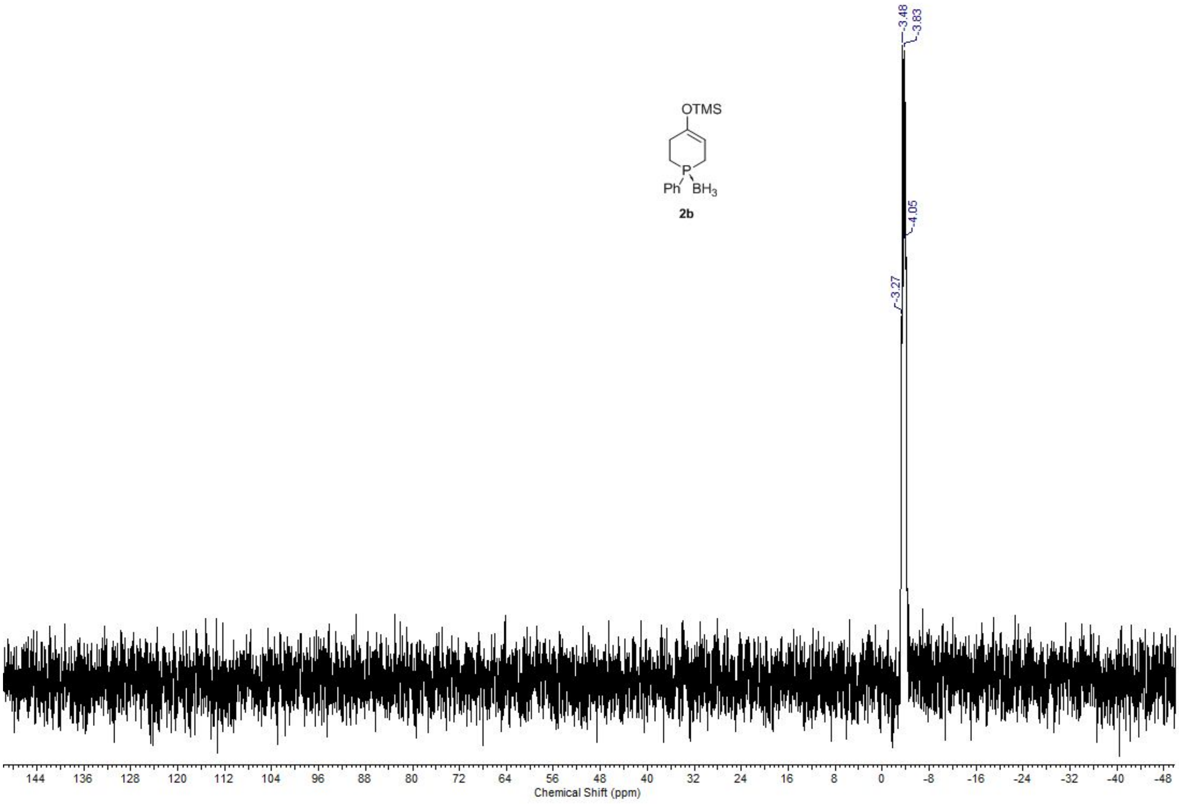

^{31}P{^{1}H} NMR spectrum of 1-phenyl-4-[(trimethylsilyl)oxy]-1,2,3,6-tetrahydrophosphinine 1-borane (**2b**) ($CDCl_3$, 202 MHz).

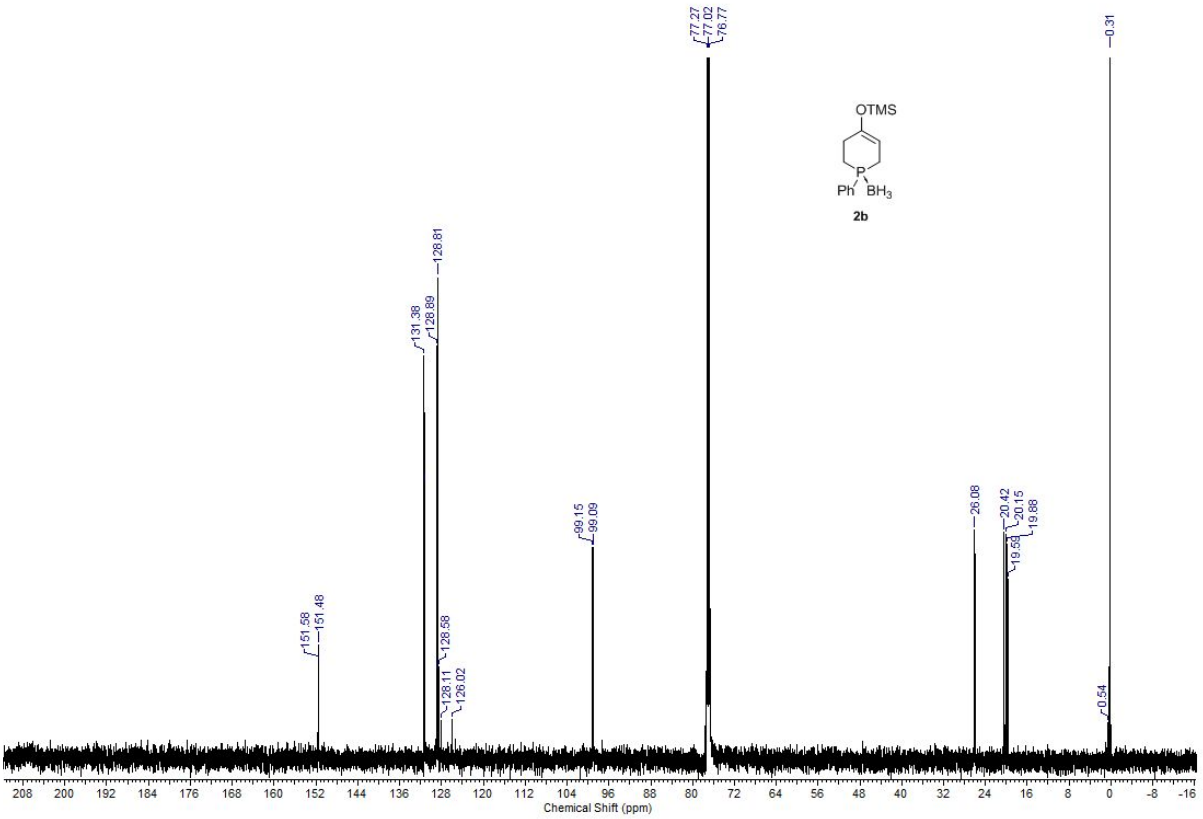

$^{31}C\{^{1}H\}$ NMR spectrum of 1-phenyl-4-[(trimethylsilyl)oxy]-1,2,3,6-tetrahydrophosphinine 1-borane (**2b**) ($CDCl_3$, 126 MHz).

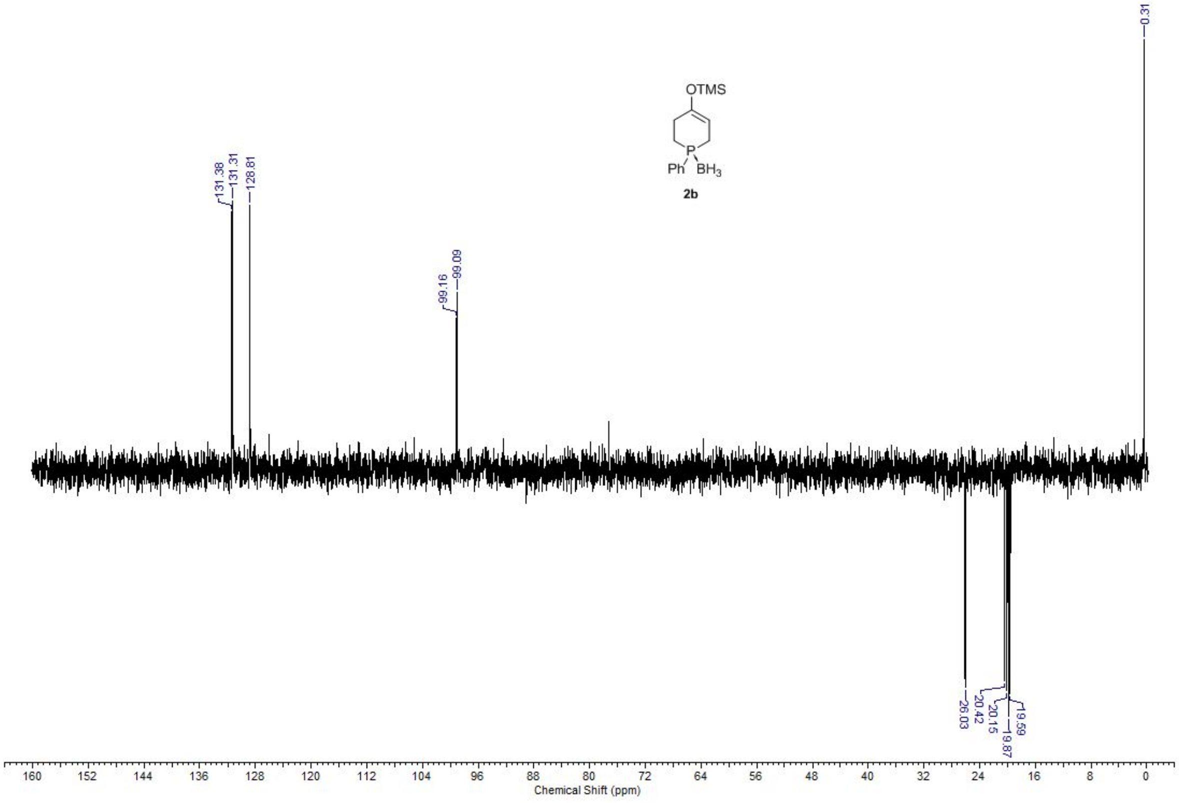

DEPT 135 spectrum of 1-phenyl-4-[(trimethylsilyl)oxy]-1,2,3,6-tetrahydrophosphinine 1-borane (**2b**) ($CDCl_3$, 126 MHz).

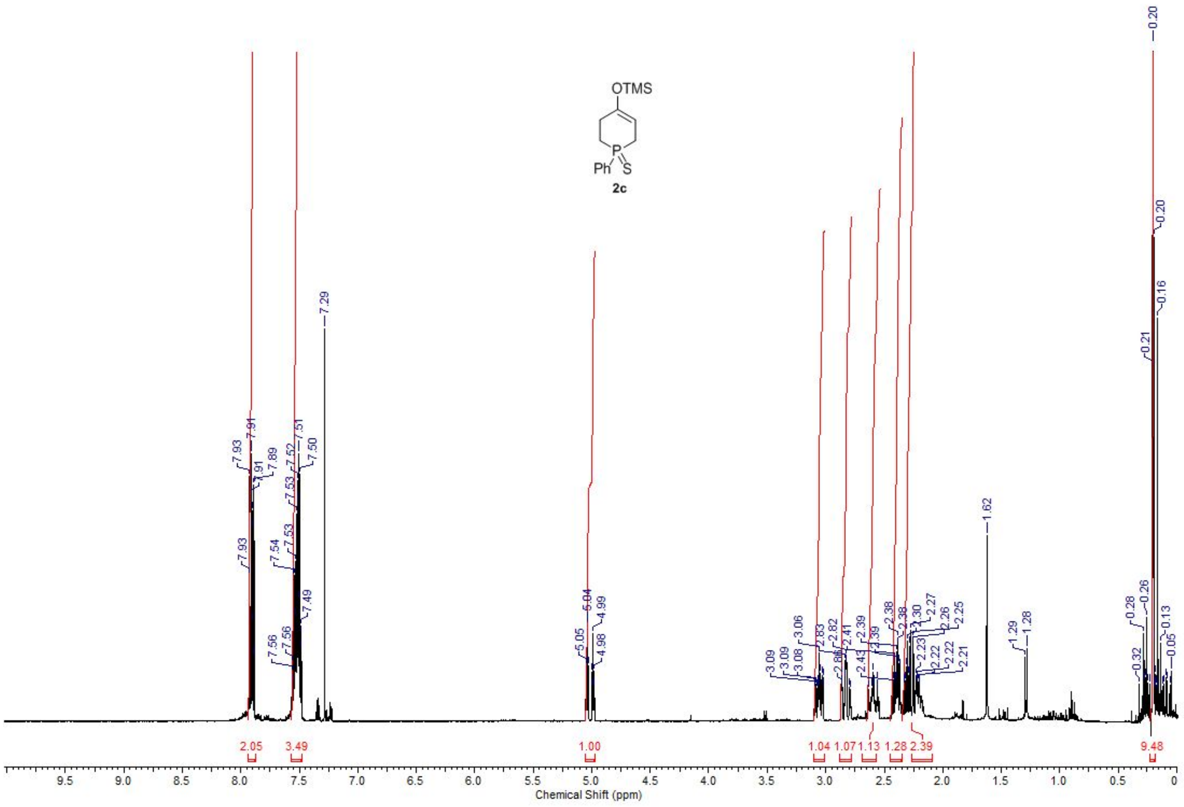

^{1}H NMR spectrum of 1-phenyl-4-[(trimethylsilyl)oxy]-1,2,3,6-tetrahydrophosphinine 1-sulfide (**2c**) ($CDCl_3$, 500 MHz).

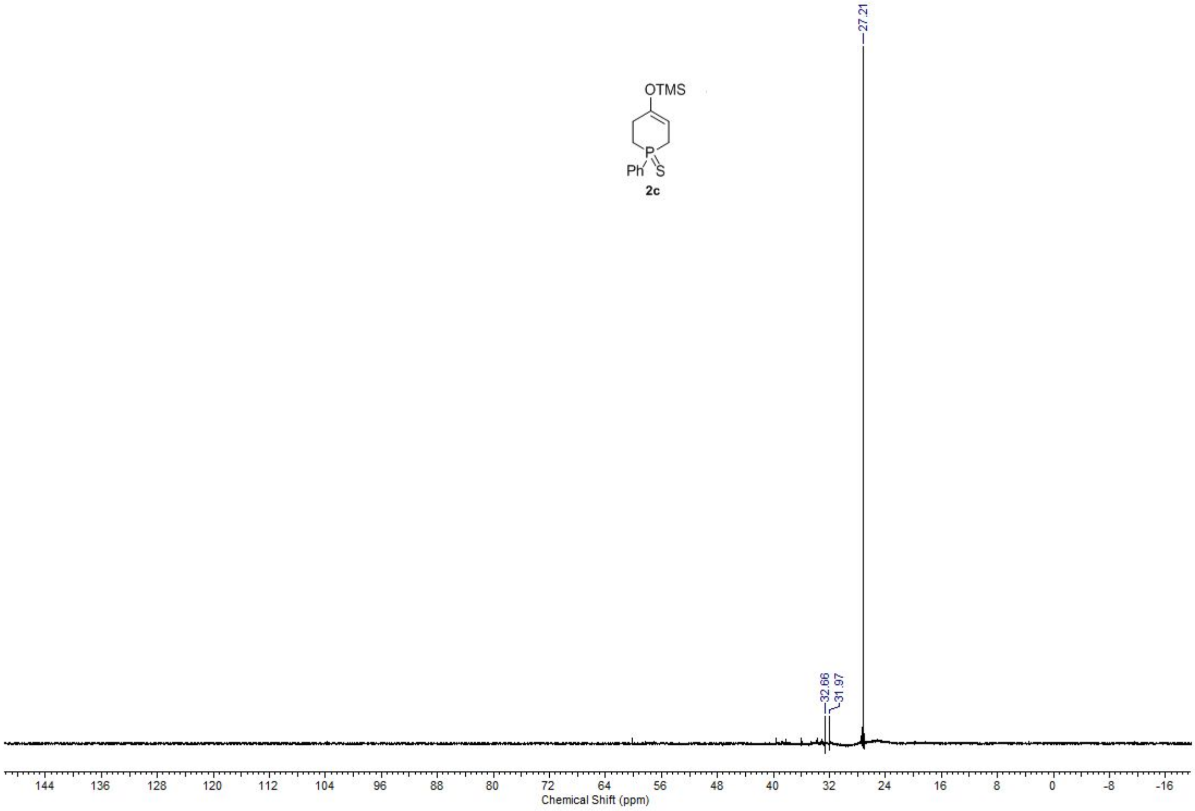

^{31}P{^{1}H} NMR spectrum of 1-phenyl-4-[(trimethylsilyl)oxy]-1,2,3,6-tetrahydrophosphinine 1-sulfide (**2c**) ($CDCl_3$, 202 MHz).

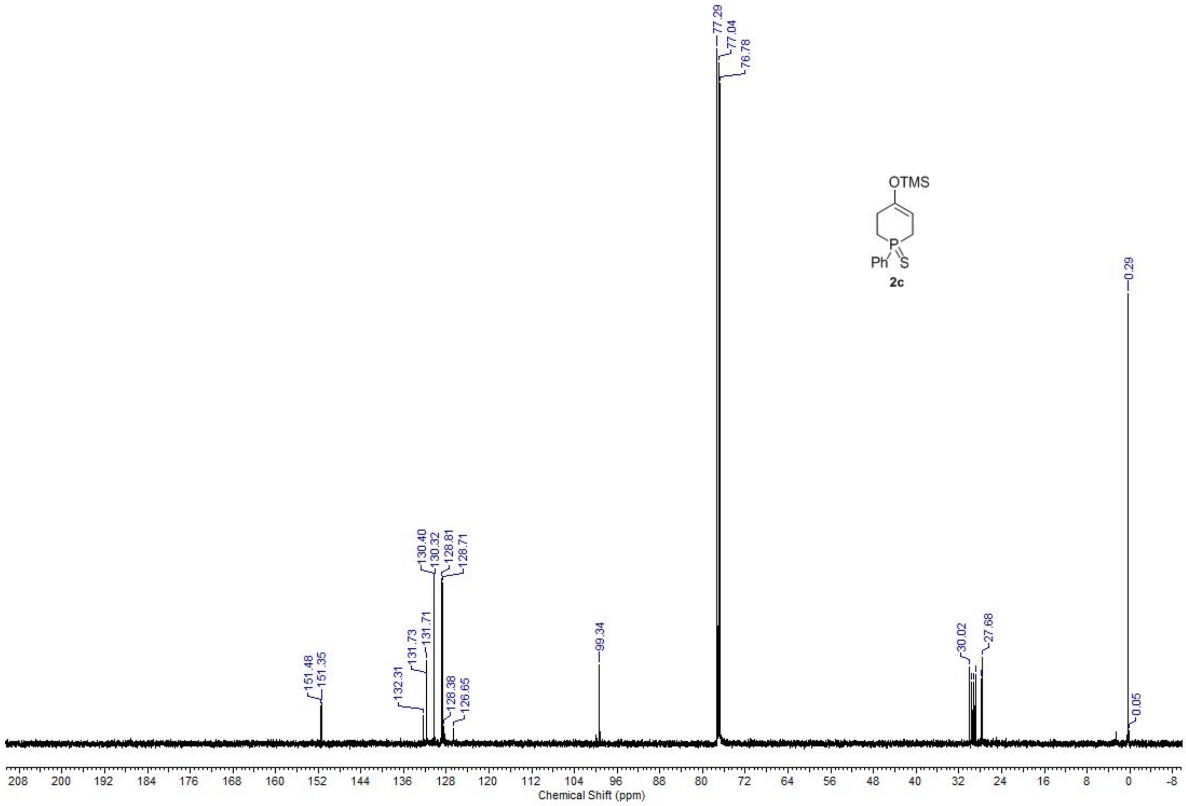

$^{31}C\{^{1}H\}$ NMR spectrum of 1-phenyl-4-[(trimethylsilyl)oxy]-1,2,3,6-tetrahydrophosphinine 1-sulfide (**2c**) ($CDCl_3$, 126 MHz).

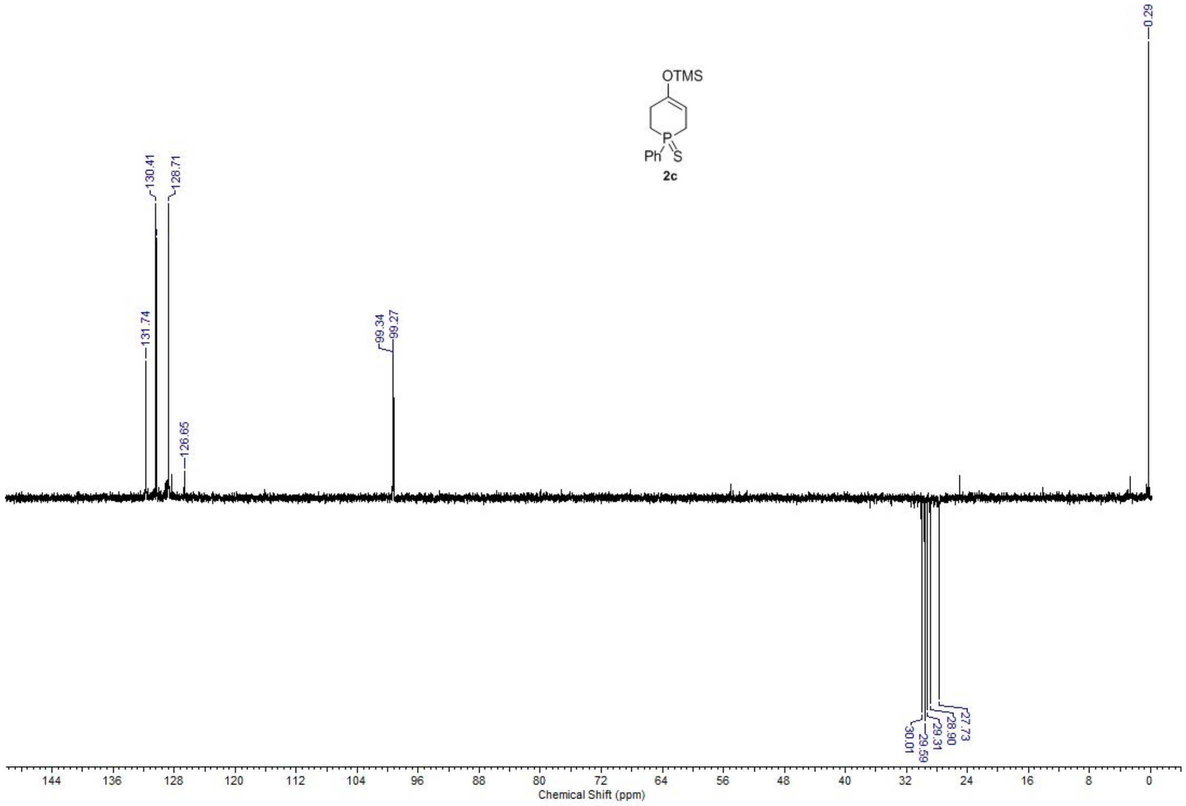

DEPT 135 spectrum of 1-phenyl-4-[(trimethylsilyl)oxy]-1,2,3,6-tetrahydrophosphinine 1-sulfide (**2c**) ($CDCl_3$, 126 MHz).

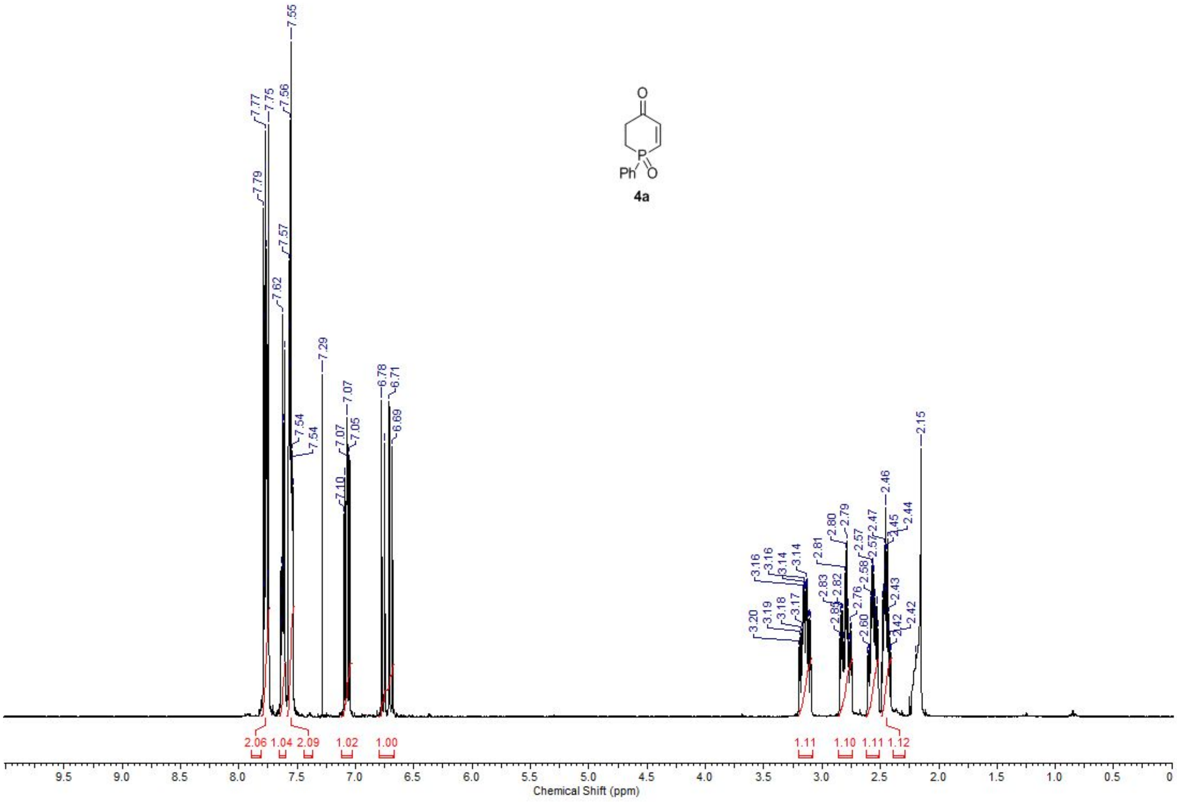

^{1}H NMR spectrum of 1-phenylphosphin-2-en-4-one 1-oxide (**4a**) ($CDCl_3$, 500 MHz).

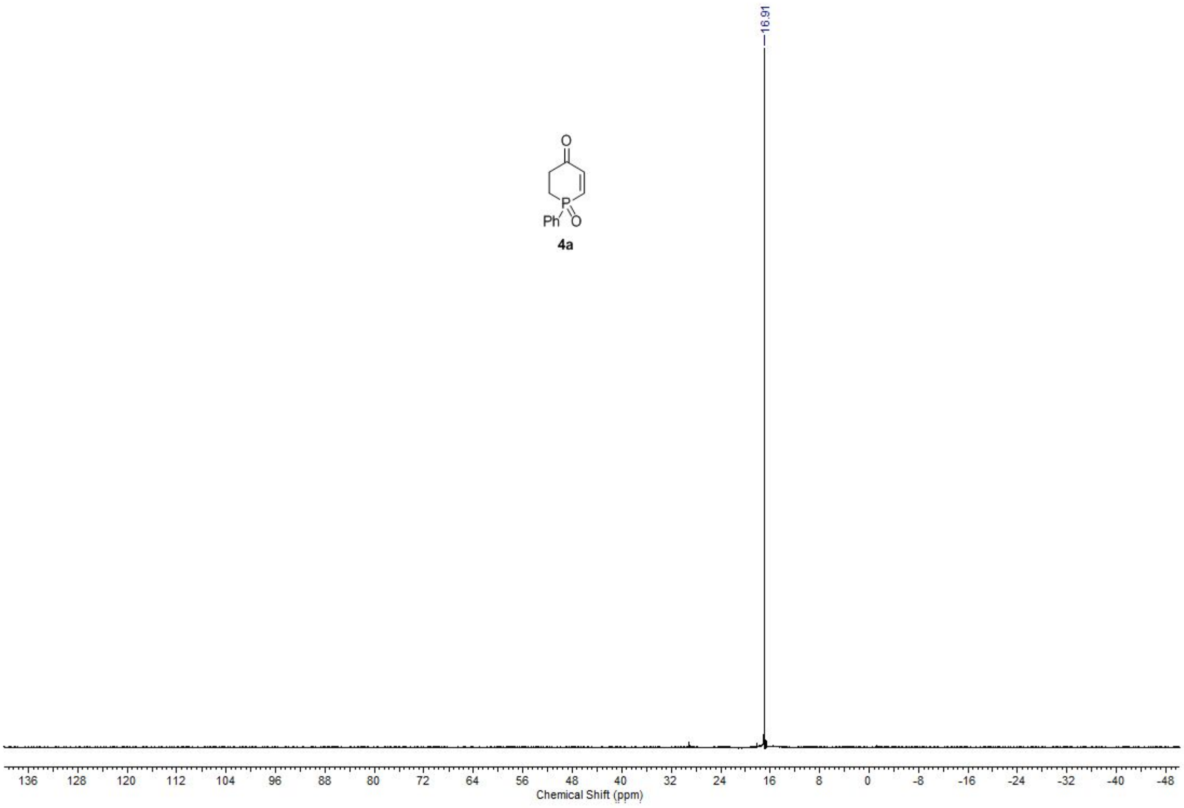

$^{31}P\{^{1}H\}$ NMR spectrum of 1-phenylphosphin-2-en-4-one 1-oxide (**4a**) ($CDCl_3$, 202 MHz).

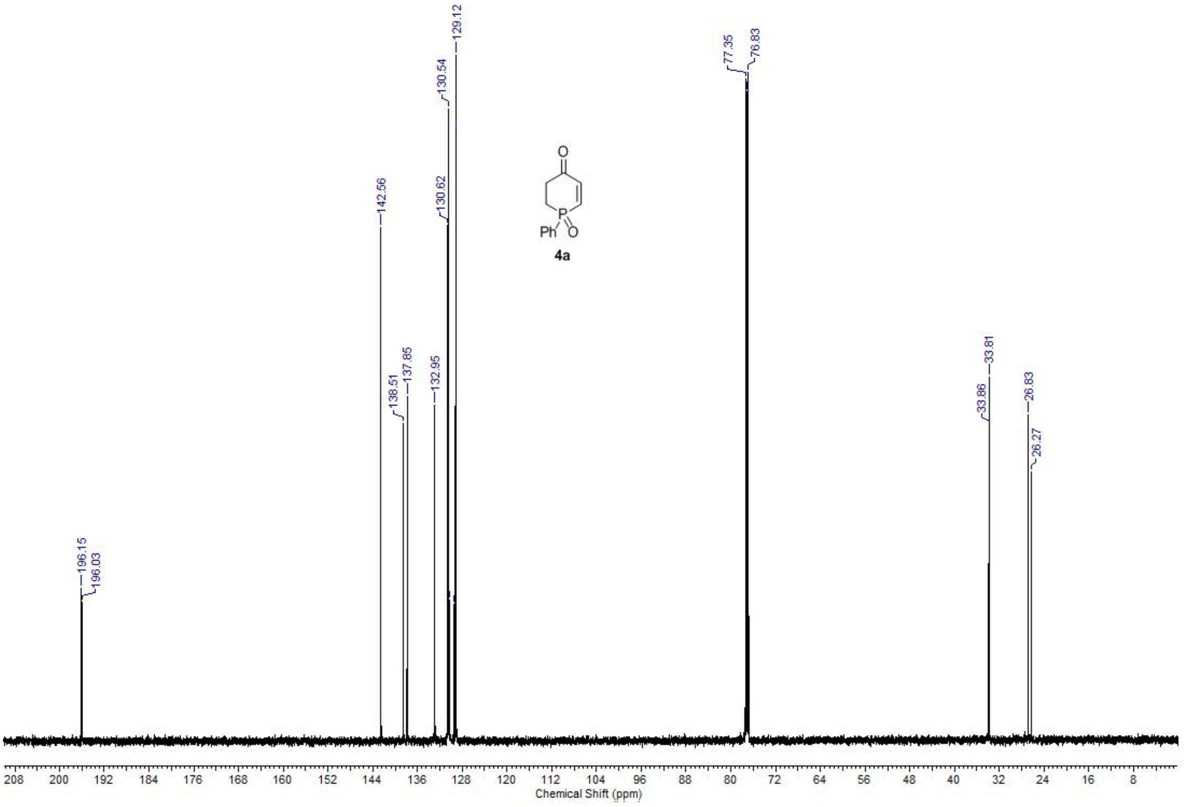

$^{31}C\{^{1}H\}$ NMR spectrum of 1-phenylphosphin-2-en-4-one 1-oxide (**4a**) ($CDCl_3$, 126 MHz).

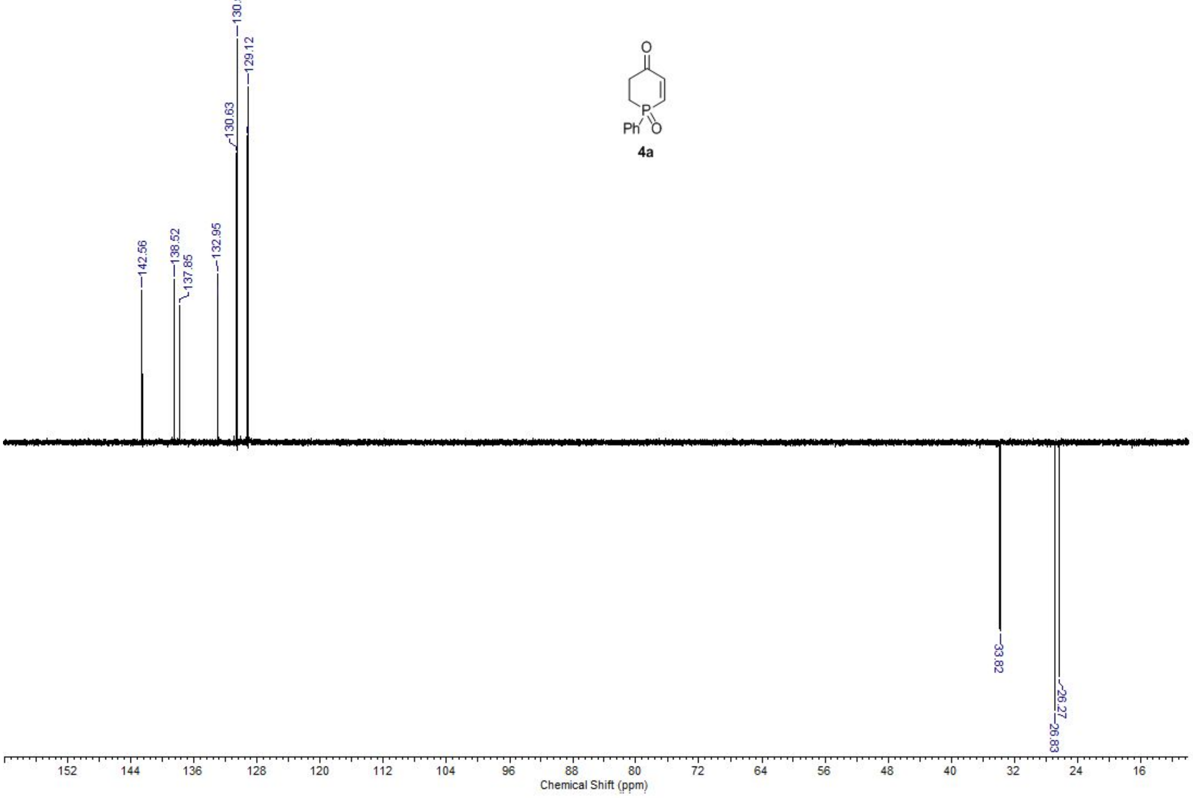

DEPT 135 spectrum of 1-phenylphosphin-2-en-4-one 1-oxide (**4a**) ($CDCl_3$, 126 MHz).

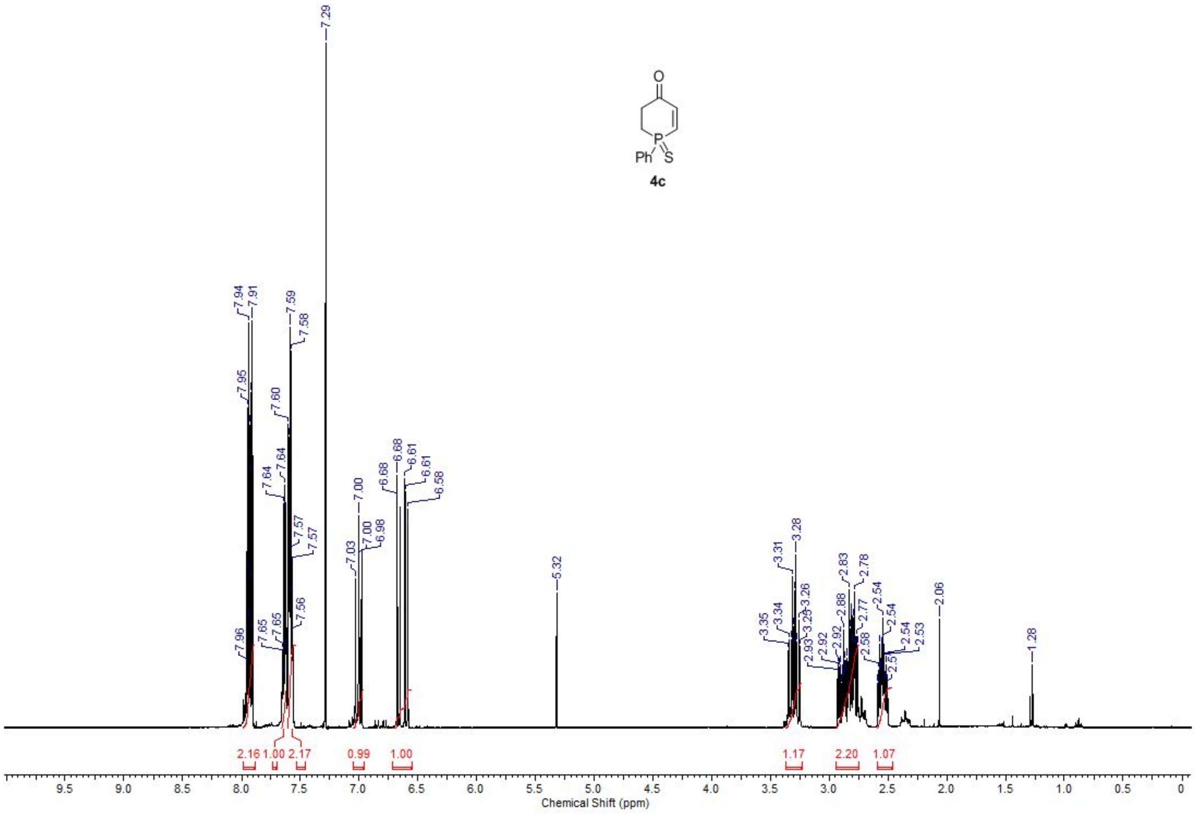

^{1}H NMR spectrum of 1-phenylphosphin-2-en-4-one 1-sulfide (**4c**) ($CDCl_3$, 500 MHz).

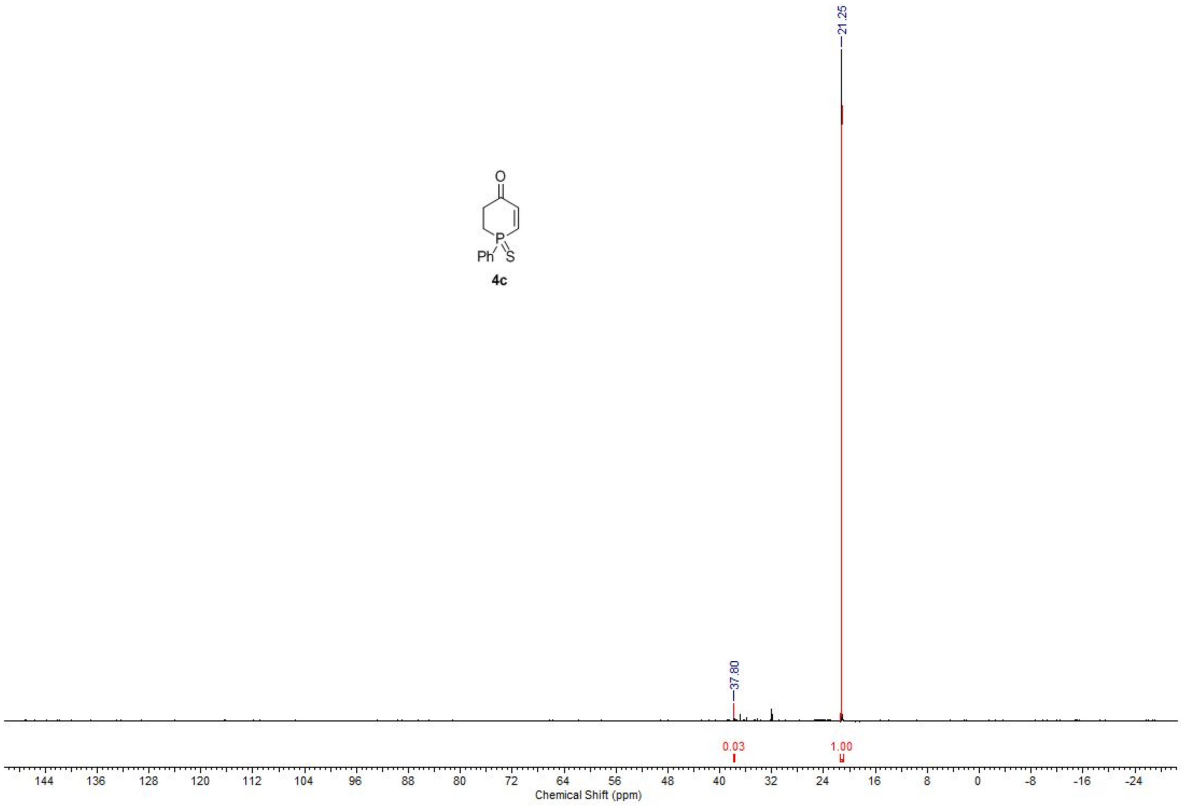

^{31}P{^{1}H} NMR spectrum of 1-phenylphosphin-2-en-4-one 1-sulfide (**4c**) ($CDCl_3$, 202 MHz).

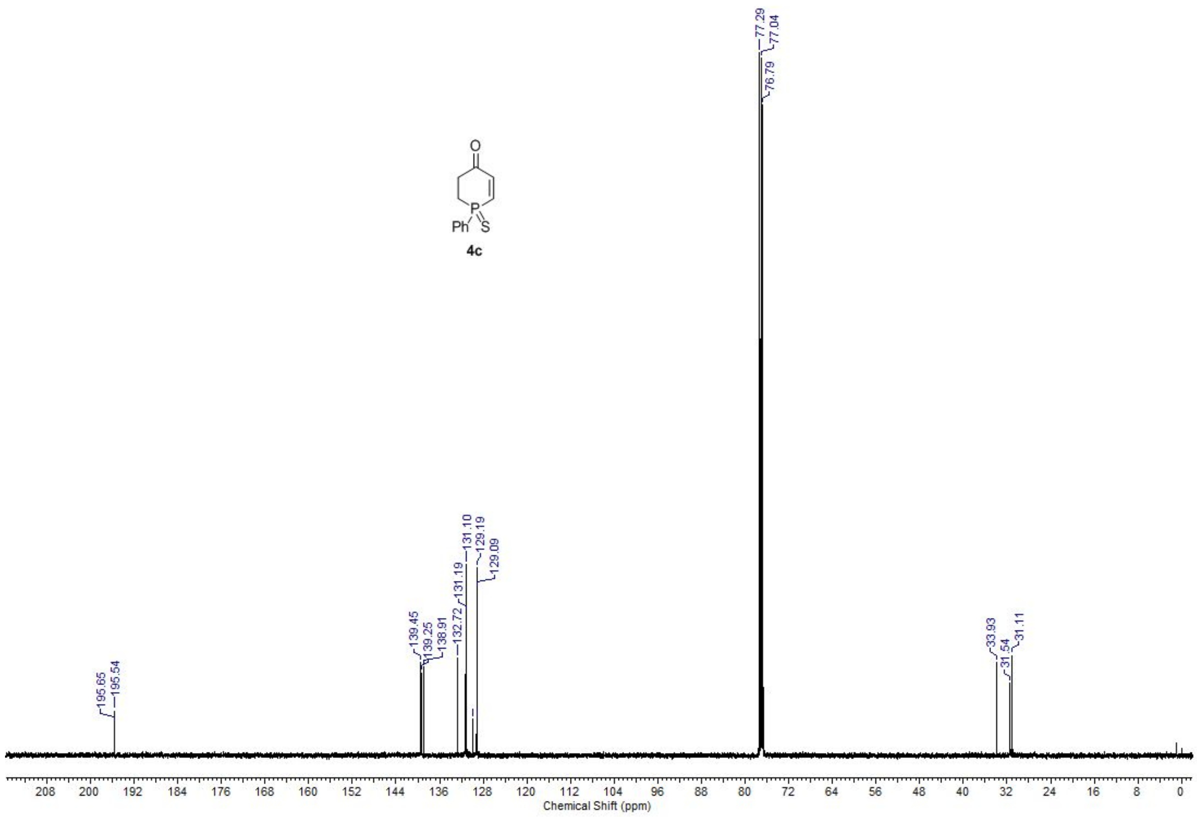

^{31}C{^{1}H} NMR spectrum of 1-phenylphosphin-2-en-4-one 1-sulfide (**4c**) ($CDCl_3$, 126 MHz).

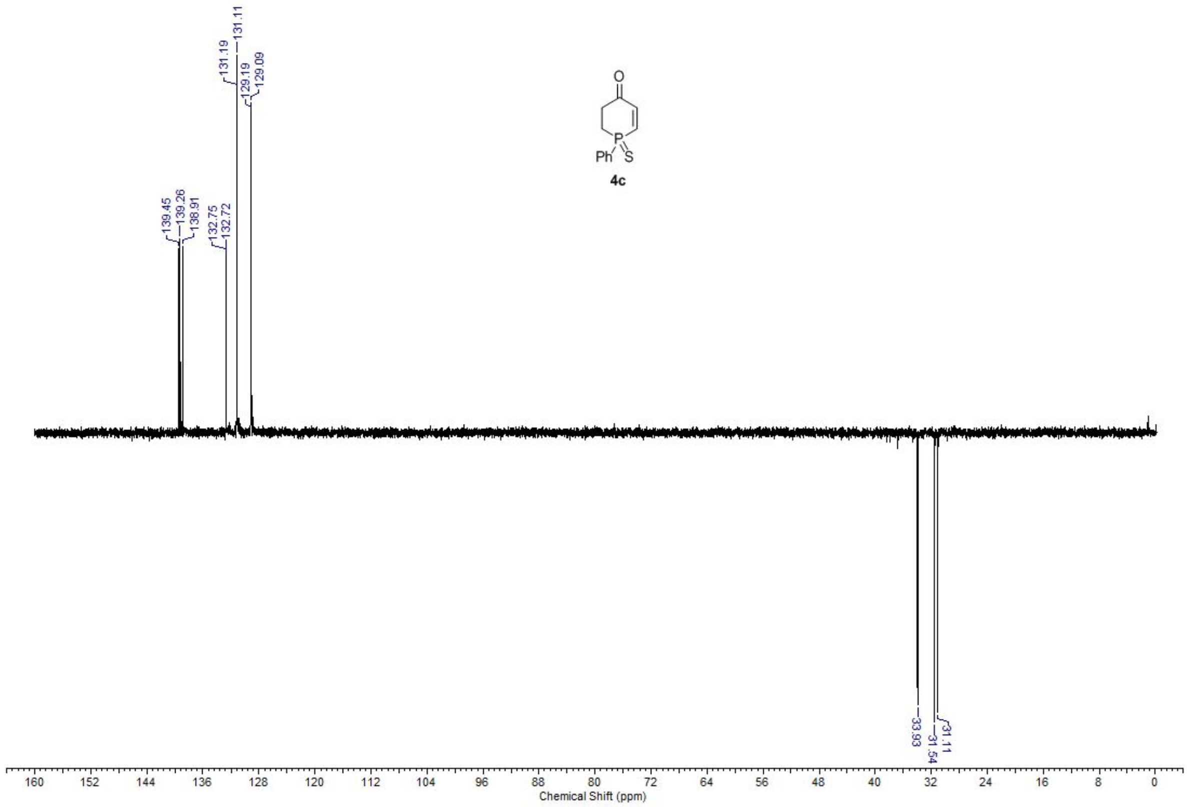

DEPT 135 spectrum of 1-phenylphosphin-2-en-4-one 1-sulfide (**4c**) ($CDCl_3$, 126 MHz).

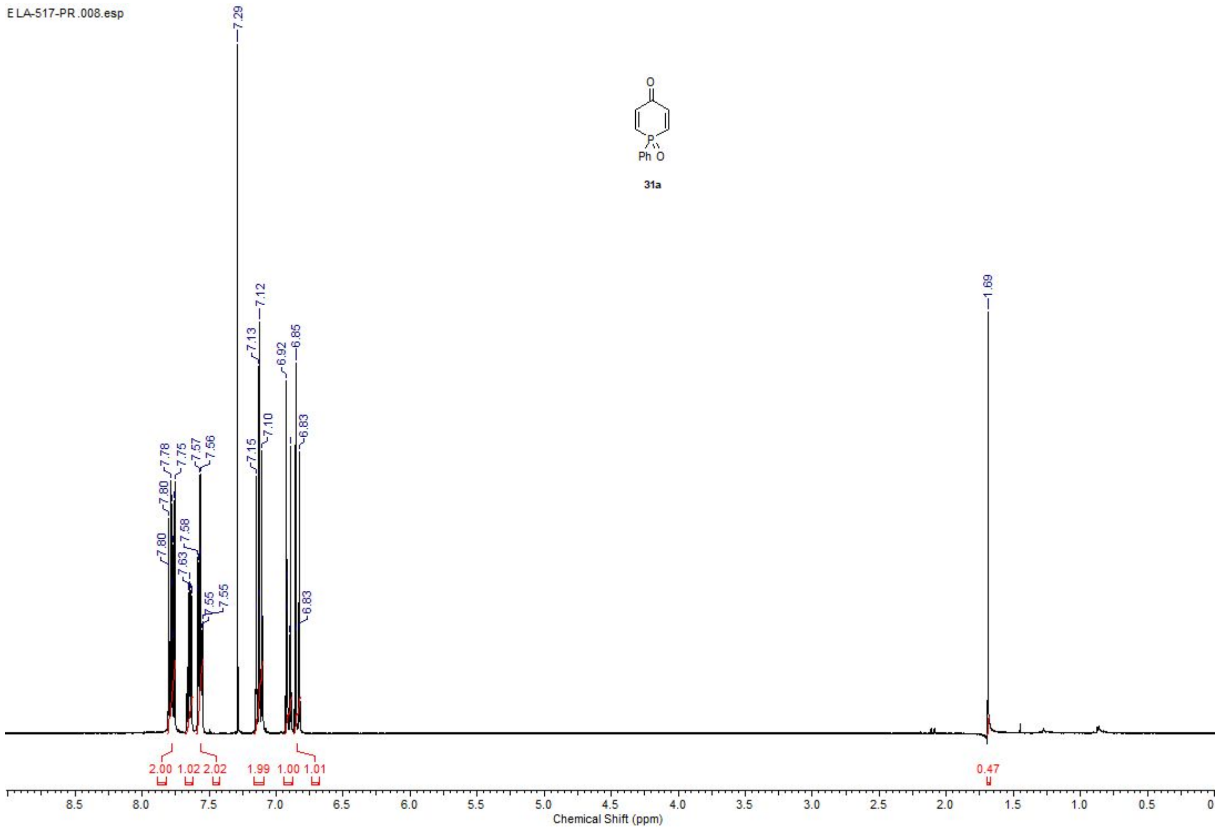

^{1}H NMR spectrum of 1-phenylphosphin-2,5-dien-4-one 1-oxide (**31a**) ($CDCl_3$, 500 MHz).

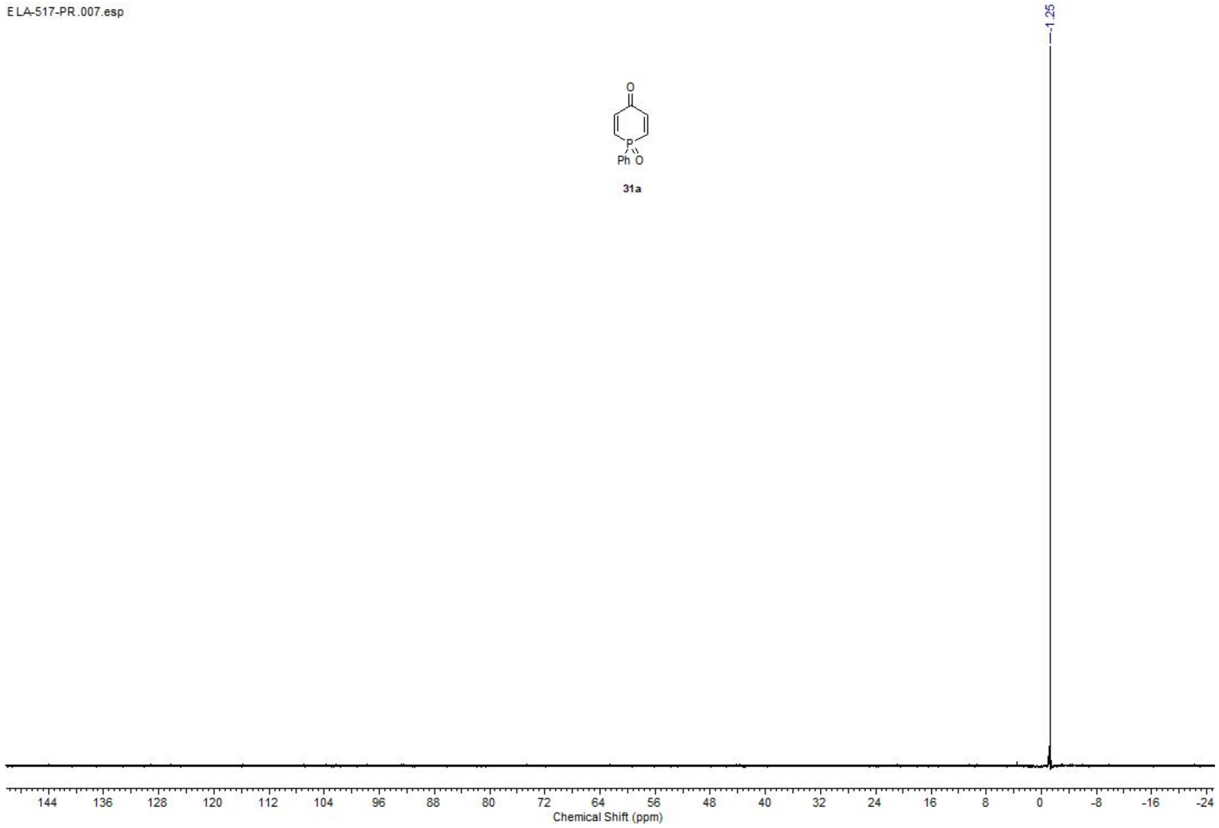

^{31}P{^{1}H} NMR spectrum of 1-phenylphosphin-2,5-dien-4-one 1-oxide (**31a**) ($CDCl_3$, 202 MHz).

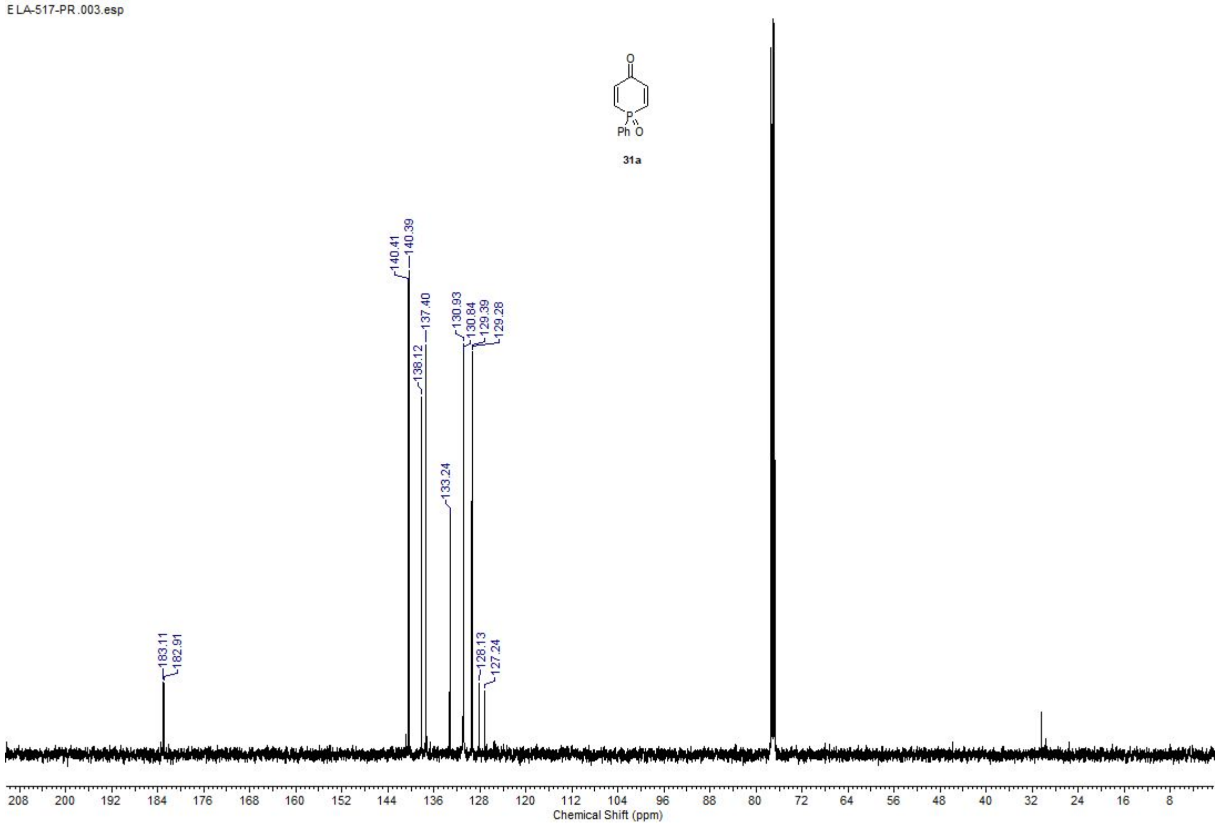

^{31}C{^{1}H} NMR spectrum of 1-phenylphosphin-2,5-dien-4-one 1-oxide (**31a**) ($CDCl_3$, 126 MHz).

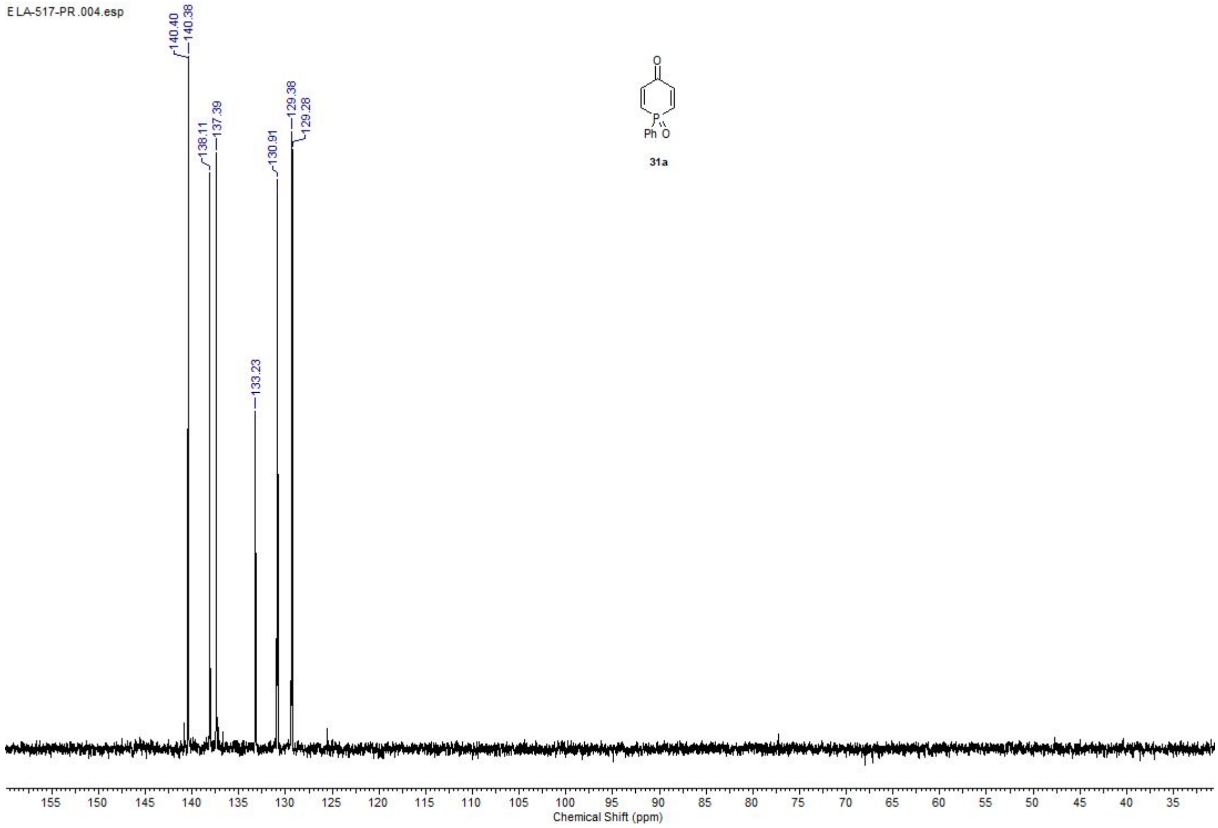

DEPT 135 spectrum of 1-phenylphosphin-2,5-dien-4-one 1-oxide (**31a**) ($CDCl_3$, 126 MHz).
